# Social deprivation and morbidity and mortality after surgery: a UK national observational cohort study

**DOI:** 10.1016/j.bja.2025.07.058

**Published:** 2025-09-23

**Authors:** Eimear Lusby, Danny J.N. Wong, Ruaraidh Campbell, Bo Hou, Snehal Pinto Pereira, SNAP-2: EpiCCS collaborators, Steve K. Harris, S. Ramani Moonesinghe

**Affiliations:** 1 Centre for Research and Improvement, Royal College of Anaesthetists, London, UK; 2 Department of Targeted Intervention, University College London, London, UK; 3 Department of Anaesthesiology, Singapore General Hospital, Singapore; 4 Department of Surgical and Interventional Engineering, Faculty of Life Sciences & Medicine, King's College London, London, UK; 5 Institute of Health Informatics, University College London, London, UK; 6 National Institute for Health Research, Central London Patient Safety Research Collaboration, London, UK; 7 National Institute for Health Research, University College London Hospitals Biomedical Research Centre, University College, London, UK

**Keywords:** healthcare disparities, morbidity, perioperative care, postoperative complications, socioeconomic factors, surgery

## Abstract

**Background:**

Socioeconomic deprivation is associated with poor surgical outcomes. We assessed associations between deprivation and postoperative morbidity and mortality in a UK-wide surgical cohort.

**Methods:**

We analysed UK data from the Second Sprint National Anaesthesia Project: Epidemiology of Critical Care provision after Surgery (SNAP-2: EpiCCS), a prospective non-consenting cohort study of adults undergoing elective and emergency inpatient noncardiac surgery. Socioeconomic deprivation was reported using the standardised aggregate scale, Index of Multiple Deprivation (IMD; IMD1: most deprived, IMD5: least deprived). Multivariable mixed effects logistic regression was used to model the association between deprivation and postoperative outcomes, adjusting for potential confounders.

**Results:**

Of the 18 901 patients included, those in more deprived groups were younger, had higher disease prevalence, and had greater illness severity. Morbidity, as measured by the Post-Operative Morbidity Survey, was reported in 13.7% at day 7, and in-hospital 30-day mortality was 1.3%. Adjusting for patient characteristics and surgical factors, the odds ratios (ORs) for morbidity at day 7 were 1.26 (95% confidence interval [95% CI]: 1.09–1.47) for IMD2 and 1.32 (95% CI: 1.13–1.53) for IMD1, compared with IMD5. Mortality risk was also higher: OR 1.75 (95% CI: 1.12–1.73) for IMD2 and OR 1.90 (95% CI: 1.22–2.95) for IMD1. However, after adjusting for markers of preoperative physical status and comorbidities, the association between deprivation and outcomes was attenuated.

**Conclusions:**

Socioeconomic deprivation is associated with short-term postoperative morbidity and mortality. This association might relate to poorer baseline fitness among people living in socioeconomically deprived areas, highlighting opportunities for targeted preoperative optimisation.

**Editor’s key points**

• Associations between social deprivation and postoperative morbidity and mortality were analysed using SNAP-2: EpiCCS data, a prospective non-consenting UK-wide cohort study of adults undergoing elective and emergency inpatient noncardiac surgery.
• Postoperative morbidity and mortality were more common in patients from more deprived postcodes, but this association was attenuated after adjusting for markers of preoperative physical status and comorbidities.
• This association with social deprivation might be related to poorer baseline fitness, highlighting opportunities for targeted preoperative optimisation to reduce disparities in surgical outcomes.

Socioeconomic deprivation is strongly linked to adverse health outcomes, including increased risk of disease, delayed diagnoses, and reduced life expectancy.^1^, ^2^, ^3^ This relationship is also observed in outcomes after surgery, where deprivation is associated with increased risk of perioperative complications, prolonged length of stay, and death.^4^, ^5^, ^6^, ^7^, ^8^ This association is present in countries with universal access healthcare systems and those with fee-based systems alike.^9^^,^^10^

Previous research has primarily focused on the impact of social deprivation on mortality. Morbidity might be a more sensitive measure for detecting a difference between groups, as mortality is a rare outcome from major elective surgery, with <1% risk globally.^11^ Postoperative morbidity could also be a more clinically relevant measure than in-hospital mortality, given its impact on quality of life^12^ and its link to reduced long-term survival.^13^^,^^14^ In addition, morbidity drives length of stay and has significant cost implications^15^ for a healthcare service.

Where non-mortality outcomes have been studied in the context of socioeconomic deprivation, most research has been conducted in the USA and other countries with fee-based or insurance-based healthcare systems,^4^, ^5^, ^6^ where limited access to healthcare can exclude the most deprived groups. In contrast, the UK National Health Service (NHS), which provides free access at the point of delivery, offers a unique setting to explore the association between deprivation and surgical outcomes. The impact of social deprivation on postoperative morbidity in the UK remains underexplored.

Most research in this area involves retrospective analysis of administrative databases, which often lack detailed information on perioperative risk factors and complications. Although prospective studies address this, they can be adversely affected by recruitment bias, as those who participate in research tend to be of higher socioeconomic status, with lower rates of disease burden and mortality compared with non-responders.^16^ Our analysis uses prospectively collected data from a heterogeneous UK-wide surgical cohort, with a non-consenting study design that minimises recruitment bias and enhances the reliability of our findings. This study aimed to assess the association between socioeconomic deprivation and postoperative morbidity, in a population-representative heterogeneous cohort. The secondary objective is to evaluate the association between deprivation and 30-day mortality.

## Methods

### Study cohort

This is a *post hoc* analysis of prospectively collected data from the Second Sprint National Anaesthesia Project: Epidemiology of Critical Care provision after Surgery (SNAP-2: EpiCCS), linked with national audit databases. SNAP-2: EpiCCS was a prospective cohort study of adults undergoing inpatient surgery in the UK, Australia, and New Zealand aimed at evaluating risk factors for poor surgical outcomes, the validity of clinical risk scores, and access to postoperative critical care. Full inclusion criteria of SNAP-2: EpiCCS have been described.^17^ All adults undergoing inpatient emergency and elective surgery between March 21 and 27, 2017, were included in this analysis in the UK subcohort only, owing to availability of linked deprivation data. Patients undergoing obstetric surgery were excluded *a priori*, as this was a very low-risk population.

SNAP-2: EpiCCS was a non-consenting study approved by the Health Research Authority (South Central–Berkshire B REC, reference number: 16/SC/0349), with a Section 251 exemption to collect patient-identifiable information to enable linkage of prospectively collected data with external databases. Ethical approval for this analysis of anonymised data was not required. We report our findings in accordance with the Strengthening the Reporting of Observational Studies in Epidemiology guidance.^18^

### Measurement of deprivation

Deprivation was measured using the Index of Multiple Deprivation (IMD), a composite score published by the UK Office of National Statistics (ONS) that ranks areas based on their relative level of deprivation.^19^ The score is based on 37 different indicators grouped into seven broad domains: income, employment, education, health, crime, barriers to housing and services, and the living environment. These indicators are aggregated to generate a score for each lower-layer super output area (LSOA), a small area with an average of 1500 residents. Patients were assigned an IMD quintile by linking post code data, collected prospectively, to the LSOAs, and the IMD scores were then grouped into quintiles. This was derived from a publicly available ONS database from 2017.^20^ Participants in IMD1 lived in areas classified as the most deprived, and those in IMD5 in the least deprived areas.

### Outcome measures

The primary outcome, postoperative morbidity, was present if a patient remained in hospital 7 days after operation and had Post-Operative Morbidity Survey (POMS)-major defined morbidity recorded in the preceding 24 h. POMS is a validated tool designed to detect short-term morbidity after surgery that requires a patient to remain in hospital,^21^ and POMS-major morbidity, a subset of the POMS criteria, represents clinically significant morbidity.^22^ A full list of POMS criteria is available in Supplementary Table S1. As the mean length of stay in the UK surgical population is 6 days,^23^ a postoperative stay of 7 days is likely indicative of patients with ongoing morbidity. Patients discharged before day 7 were assumed to have no major morbidity, consistent with previous studies.^21^ Patients who died before day 7 were treated as having morbidity. The secondary outcome was in-hospital 30-day mortality, recorded prospectively by local SNAP-2 investigators.

### Data sources and linkage

Data for this analysis was collected prospectively by local SNAP-2: EpiCCS investigators, with the exception of IMD and ethnicity, which was derived from linked Hospital Episode Statistics data.

### Statistical analysis

A statistical analysis plan was developed *a priori* before accessing the SNAP-2: EpiCCS dataset. Descriptive statistics for baseline characteristics of patients across IMD quintiles are presented as proportions and medians and interquartile ranges (IQRs) as appropriate, and compared using Pearson’s χ^2^ test for categorical variables, and Kruskal–Wallis test for continuous variables.

Cases with missing or duplicate anonymised NHS numbers and cases with missing outcome data were excluded from analysis. Implausible values (e.g. BMI <14 or >70 kg m^−2^ and haemoglobin [Hb] <25 or >200 g L^−1^) were treated as missing. Some categorical variables were collapsed to avoid small cell sizes in analysis. Multiple imputation using chained equations was used to predict missing variables (40 complete data sets), following current guidelines,^24^ with the assumption that the data were missing at random (MAR). Imputation models included both predictors of missingness and variables used in the multivariable analysis to strengthen the MAR assumption; details of variables used are provided in Supplementary Table S4.

Logistic regression models were used to estimate the odds ratios (ORs) for outcomes across IMD quintiles, using IMD5 as the reference category. Univariable associations between IMD and outcomes were first assessed using single-level logistic regression. The intraclass correlation coefficient (ICC) was calculated to assess the influence of hospital clustering on outcomes; details are provided in the Supplementary material. For multivariable analysis, mixed effects logistic regression models were used, initially adjusting for patient characteristics and surgical factors only, followed by a full model including comorbidity and functional status variables, with a random intercept for hospital. This approach allowed for examination of the contribution of each set of variables to differences in outcomes across IMD quintiles. Regression analyses were performed on each complete imputed data set, and the results were pooled.

Confounding variables included in the multivariable models were selected based on their association with postoperative morbidity, or based on published risk factors.^22^^,^^25^ A full list of these variables can be found in Supplementary Table S2.

All analysis was performed using R version 4.3.1 (R Foundation for Statistical Computing, Vienna, Austria).

### Sensitivity analyses

A complete case analysis was performed as a sensitivity analysis to test the robustness of results to assumptions about missing data. Given differences in age distribution across IMD quintiles in this cohort, the analysis was also repeated in a younger sample, excluding individuals more than 70 yr of age.

## Results

**Fig 1:**
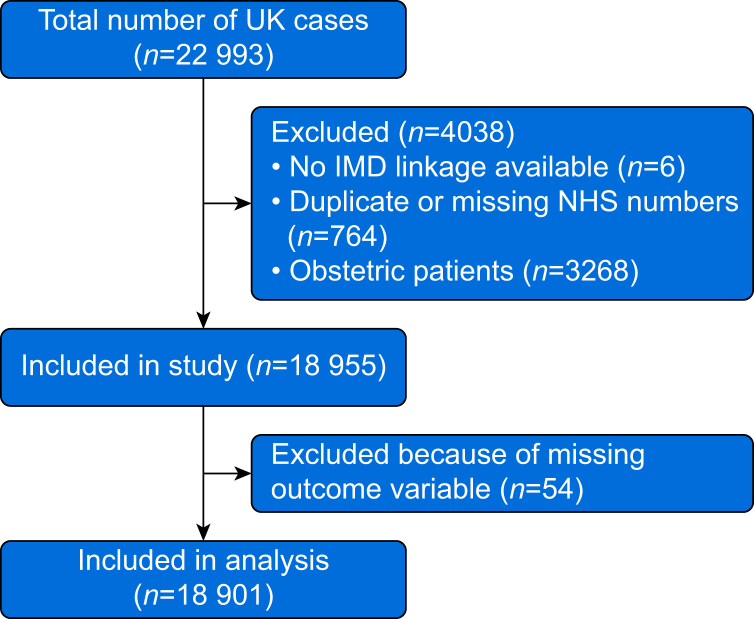
STROBE flow diagram. IMD, Index of Multiple Deprivation; STROBE, Strengthening the Reporting of Observational Studies in Epidemiology.

In total, there were 22 993 patients across 240 hospital sites in the data set. Details of the exclusions are shown in Figure 1. During this process, six patients whose main residence was in the Isle of Man and Channel Islands were excluded, as IMD scores were not available for these areas. A total of 18 901 complete cases were included in the final analysis. Overall data completeness was 97.8%. The proportion of missing data for each predictor variable was <0.5%, except for BMI (38.6%), Hb (9.8%), and IMD (6%). See Supplementary Table S3 for full missing variable analysis.

Baseline patient characteristics for the complete cases across IMD quintiles are summarised in Table 1. There were notable differences across IMD quintiles. Patients in the most deprived quintile (IMD1) were on average 10 yr younger than those in the least deprived quintile (IMD5), with a gradient of increasing age across quintiles as deprivation decreased. A higher proportion of patients from minority ethnic groups (11.6% IMD1 *vs* 3.6% IMD5) and with long-term conditions previously associated with socioeconomic deprivation, such as type 2 diabetes mellitus (14.6% IMD1 *vs* 10.2% IMD5) and chronic obstructive pulmonary disease (11.6% IMD1 *vs* 5.8% IMD5), were observed in the most deprived quintiles. There was also higher illness severity and functional impairment in the most deprived categories, with a greater proportion classified as ASA physical status 3 and above (35.5% IMD1 *vs* 29.9% IMD5). A larger percentage of patients with diabetes mellitus required insulin therapy (3.6% IMD1 *vs* 2. 2% IMD5), and severity of dyspnoea was greater: 9.2% had dyspnoea limiting activities or at rest in IMD1, compared with 5.9% in IMD5. Patients living in more deprived areas also had a lower preoperative Hb and higher creatinine levels.

**Table 1:** Characteristics of the study population across IMD quintiles. Data are provided as median (IQR) or *n* (%). *P*-values based on χ^2^ test (for categorical) or Kruskal–Wallis test (for continuous) comparing proportions across quintiles. COPD, chronic obstructive pulmonary disease; ENT, ears, nose, and throat; Hb, haemoglobin; IMD, Index of Multiple Deprivation; IQR, interquartile range; NCEPOD, National Confidential Enquiry into Patient Outcome and Death; T1DM, type 1 diabetes mellitus; T2DM, type 2 diabetes mellitus; TIA, transient ischaemic attack.

		IMD5 (least deprived)	IMD4	IMD3	IMD2	IMD1 (most deprived)	Total	*P*
Total		3618 (20.4)	3567 (20.1)	3408 (19.2)	3497 (19.7)	3675 (20.7)	17 765	
Sex	Female	1992 (55.1)	1873 (52.5)	1829 (53.7)	1929 (55.2)	1995 (54.3)	9618 (54.1)	0.144
	Male	1626 (44.9)	1694 (47.5)	1579 (46.3)	1568 (44.8)	1680 (45.7)	8147 (45.9)	
Age (yr)		66.5 (51.0–76.0)	66.0 (50.0–76.0)	63.0 (47.0–74.0)	60.0 (44.0–73.0)	56.0 (41.0–70.0)	63.0 (47.0–74.0)	<0.001
BMI (kg m^−2^)	Healthy weight (BMI 18.5–25)	615 (27.0)	553 (24.8)	519 (24.8)	471 (21.5)	480 (21.8)	2638 (24.0)	<0.001
	Underweight (BMI <18.5)	39 (1.7)	47 (2.11)	45 (2.15)	54 (2.5)	62 (2.8)	247 (2.2)	
	Overweight (BMI 25–30)	882 (38.7)	851 (38.2)	779 (37.2)	793 (36.2)	726 (33.0)	4031 (36.7)	
	Obese (BMI 30–40)	654 (28.7)	671 (30.1)	625 (29.9)	714 (32.6)	746 (33.9)	3410 (31.0)	
	Severely obese (BMI >40)	89 (3.9)	104 (4.7)	125 (5.9)	157 (7.2)	185 (8.4)	660 (6.0)	
Ethnicity	White	3486 (96.4)	3412 (95.7)	3206 (94.1)	3199 (91.5)	3251 (88.5)	16 554 (93.2)	<0.001
	Asian	83 (2.3)	84 (2.4)	109 (3.2)	140 (4.0)	220 (6.0)	636 (3.6)	
	Black	22 (0.6)	28 (0.8)	48 (1.4)	94 (2.7)	124 (3.4)	316 (1.8)	
	Other	26 (0.7)	43 (1.2)	43 (1.3)	64 (1.8)	76 (2.1)	252 (1.4)	
ASA physical status	1	751 (20.8)	704 (19.8)	668 (19.6)	689 (19.7)	736 (20.0)	3548 (20.0)	<0.001
	2	1781 (49.3)	1682 (47.2)	1566 (46.0)	1589 (45.6)	1627 (44.3)	8245 (46.5)	
	3	931 (25.7)	993 (27.9)	1004 (29.5)	1013 (29.0)	1099 (30.0)	5040 (28.4)	
	4	141 (3.9)	175 (4.9)	161 (4.7)	194 (5.5)	197 (5.4)	868 (4.9)	
	5	11 (0.3)	7 (0.2)	5 (0.1)	7 (0.2)	9 (0.2)	39 (0.2)	
Coronary artery disease		440 (12.2)	476 (13.3)	430 (12.6)	462 (13.2)	499 (13.6)	2307 (13.0)	0.373
Congestive cardiac failure		136 (3.8)	135 (3.8)	108 (3.2)	116 (3.3)	127 (3.5)	622 (3.5)	0.546
COPD		211 (5.8)	274 (7.7)	275 (8.1)	327 (9.4)	425 (11.6)	1512 (8.5)	<0.001
Diabetes mellitus	None	3214 (88.8)	3087 (86.5)	2928 (85.9)	2975 (85.1)	3084 (83.9)	15 288 (86.1)	<0.001
	T1DM	35 (1.0)	42 (1.2)	46 (1.3)	52 (1.5)	53 (1.4)	228 (1.3)	
	T2DM (diet controlled)	104 (2.9)	107 (3.0)	85 (2.5)	116 (3.3)	83 (2.3)	495 (2.8)	
	T2DM (oral medication)	183 (5.1)	235 (6.6)	240 (7.0)	265 (7.6)	320 (8.7)	1243 (7.0)	
	T2DM (insulin controlled)	79 (2.2)	92 (2.6)	104 (3.1)	87 (2.5)	131 (3.6)	493 (2.8)	
Cancer (current or last 5 yr)		611 (16.9)	576 (16.2)	502 (14.7)	482 (13.8)	461 (12.6)	2632 (14.8)	<0.001
Kidney disease		40 (1.1)	47 (1.3)	45 (1.3)	57 (1.6)	66 (1.8)	255 (1.4)	0.102
Cirrhosis		20 (0.6)	25 (0.7)	29 (0.9)	33 (0.9)	50 (1.4)	157 (0.9)	0.004
Stroke/TIA		208 (5.7)	202 (5.7)	230 (6.7)	181 (5.2)	215 (5.9)	1036 (5.8)	0.084
Level of dyspnoea before surgery	None	2920 (81.1)	2750 (77.4)	2659 (78.4)	2664 (76.5)	2760 (75.3)	13 753 (77.7)	<0.001
	On exertion	466 (12.9)	527 (14.8)	518 (15.3)	521 (15.0)	566 (15.4)	2598 (14.7)	
	Limiting activities or at rest	214 (5.9)	274 (7.7)	216 (6.4)	299 (8.6)	339 (9.2)	1342 (7.6)	
Anaemia before surgery	No anaemia (Hb >130 g L^-1^)	1987 (60.7)	1924 (59.4)	1846 (60.1)	1895 (60.0)	1831 (55.8)	9483 (59.2)	<0.001
	Mild anaemia (Hb 110–129 g L^-1^)	954 (29.1)	929 (28.7)	861 (28.0)	869 (27.5)	942 (28.7)	4555 (28.4)	
	Moderate anaemia (Hb 80–109 g L^-1^)	318 (9.7)	352 (10.9)	350 (11.4)	369 (11.7)	462 (14.1)	1851 (11.5)	
	Severe anaemia (Hb <80 g L^-1^)	17 (0.5)	33 (1.0)	17 (0.6)	25 (0.8)	49 (1.5)	141 (0.9)	
Preoperative creatinine	Normal (40-90 μM in females, 60-110 μM in males	2637 (81.9)	2593 (81.1)	2390 (79.2)	2441 (79.5)	2490 (78.1)	12 551 (80.0)	<0.001
	Low (<40 μM in females, <60 μM in females)	131 (4.1)	127 (4.0)	156 (5.2)	168 (5.5)	215 (6.7)	797 (5.1)	
	High (>110 μM in males, > 90 μM in females	451 (14.0)	476 (14.9)	471 (15.6)	462 (15.0)	483 (15.2)	2343 (14.9)	
Procedure severity (based on AXA specialist procedure codes)	Minor or Intermediate	865 (23.9)	896 (25.1)	897 (26.3)	988 (28.3)	1096 (29.8)	4742 (26.7)	<0.001
	Major	1240 (34.3)	1146 (32.1)	1128 (33.1)	1182 (33.8)	1258 (34.2)	5954 (33.5)	
	Extra-major	925 (25.6)	948 (26.6)	858 (25.2)	842 (24.1)	820 (22.3)	4393 (24.7)	
	Complex	588 (16.3)	577 (16.2)	525 (15.4)	485 (13.9)	501 (13.6)	2676 (15.1)	
Operative urgency (NCEPOD classification)	Elective	2160 (59.7)	2032 (57.0)	1900 (55.8)	1942 (55.5)	1913 (52.1)	9947 (56.0)	<0.001
	Expedited	465 (12.9)	467 (13.1)	475 (13.9)	454 (13.0)	489 (13.3)	2350 (13.2)	
	Urgent	904 (25.0)	994 (27.9)	937 (27.5)	1013 (29.0)	1172 (31.9)	5020 (28.3)	
	Immediate	88 (2.4)	74 (2.1)	96 (2.8)	88 (2.5)	101 (2.7)	447 (2.5)	
Surgical specialty	Ortho	1172 (32.4)	1181 (33.1)	1073 (31.5)	1031 (29.5)	1087 (29.6)	5544 (31.2)	<0.001
	Breast and endocrine	173 (4.8)	152 (4.3)	145 (4.3)	142 (4.1)	137 (3.7)	749 (4.2)	
	ENT and MaxFax	150 (4.1)	146 (4.1)	156 (4.6)	167 (4.8)	194 (5.3)	813 (4.6)	
	Gynaecology and urology	762 (21.1)	708 (19.8)	666 (19.5)	725 (20.7)	742 (20.2)	3603 (20.3)	
	Gastrointestinal	690 (19.1)	713 (20.0)	677 (19.9)	741 (21.2)	735 (20.0)	3556 (20.0)	
	Neuro and spine	186 (5.1)	173 (4.9)	178 (5.2)	169 (4.8)	176 (4.8)	882 (5.0)	
	Thoracic	162 (4.5)	155 (4.3)	165 (4.8)	143 (4.1)	147 (4.0)	772 (4.3)	
	Vascular	114 (3.2)	91 (2.6)	92 (2.7)	78 (2.2)	131 (3.6)	506 (2.8)	
	Other	209 (5.8)	248 (7.0)	256 (7.5)	301 (8.6)	326 (8.9)	1340 (7.5)	
Procedures performed in previous 30 days (including current surgery)	1	3281 (90.9)	3187 (89.6)	3082 (90.8)	3154 (90.3)	3258 (89.0)	15 962 (90.1)	0.034
	>1	329 (9.1)	368 (10.4)	312 (9.2)	339 (9.7)	403 (11.0)	1751 (9.9)	

Conversely, patients having surgery with a diagnosis of metastatic cancer or a history of cancer in the last 5 yr were significantly more likely to be in the least deprived group (14.6% IMD1 *vs* 16.9% IMD5). These patients were also more likely to be undergoing more complex surgery. Patients in the more deprived quintiles were more likely to undergo emergency surgery, that is, urgent or immediate (34.6% IMD1 *vs* 27.4 IMD5), or have more than one procedure on this admission (11% IMD1 *vs* 9.1% IMD5) (all *P*<0.001).

### Deprivation and postoperative morbidity

**Fig 2:**
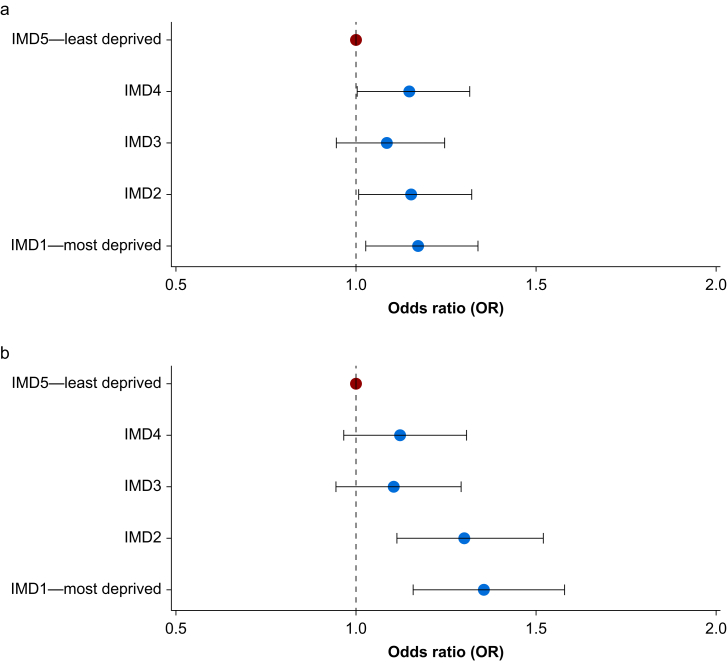
Index of Multiple Deprivation (IMD) and odds of morbidity after surgery: (a) unadjusted and (b) mixed effects model adjusted for patient characteristics and surgical factors. Model fixed effects: age, sex, ethnicity, BMI, operative urgency, procedure severity, surgical specialty, number of procedures in preceding 30 days. Red dot: IMD 5, reference level. Blue dots: OR of morbidity for each IMD. Random effect: hospital.

Postoperative morbidity as defined by POMS-major was reported in 13.7% (*n*=2592) of cases. Morbidity was present in 14.2% (*n*=522) of patients in the most deprived quintile, compared with 12.4% (*n*=448) of those in the least deprived. Patients in the two most deprived quintiles were significantly more likely to have morbidity compared with those in the least deprived quintile (OR IMD2: 1.15, 95% confidence interval [CI]: 1.01–1.34; OR IMD1: 1.17, 95% CI: 1.03–1.34). Adjusting for baseline patient characteristics and surgical factors in the mixed effects analysis strengthened this relationship (OR IMD2: 1.30, 95% CI: 1.11–1.52; OR IMD1: 1.35, 95% CI: 1.16–1.58; Fig. 2).

**Fig 3:**
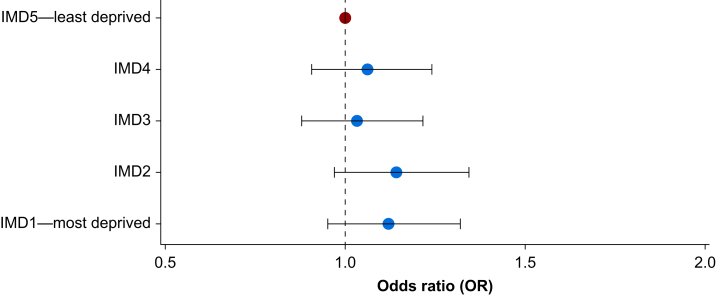
Index of Multiple Deprivation (IMD) and odds of morbidity after surgery, full multivariable mixed effects model. Model fixed effects: age, sex, ethnicity, BMI, surgical factors, ASA physical status, comorbid disease, preoperative anaemia. Red dot: IMD5, reference level. Blue dots: OR of morbidity for each IMD. Random effect: hospital.

After adjusting for predictors related to baseline physiological status, including ASA physical status, presence and severity of comorbidities, and preoperative anaemia, no overall significant association between IMD and morbidity was evident (Fig. 3). There was no evidence of multicollinearity of variables, and model fit statistics can be seen in Supplementary Table S6.

### Deprivation and postoperative mortality

**Fig 4:**
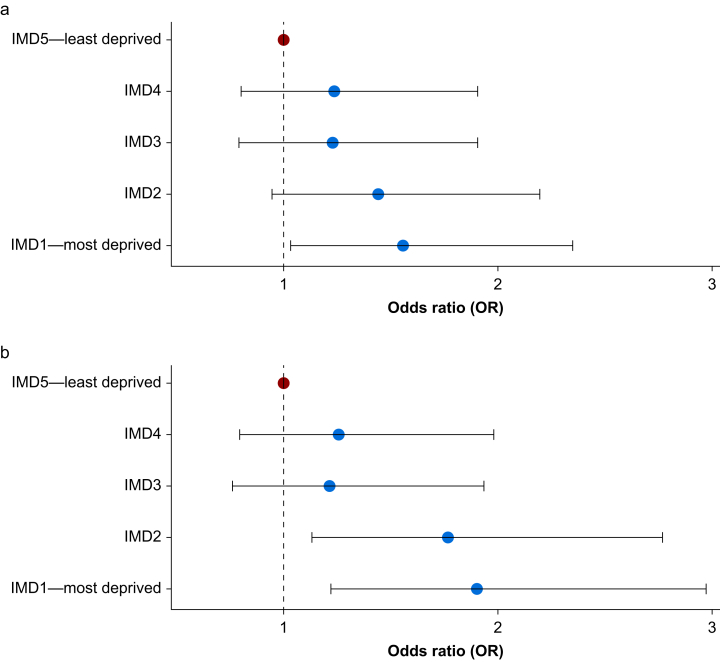
Index of Multiple Deprivation (IMD) and odds of 30-day mortality after surgery: (a) unadjusted and (b) mixed effects model adjusted for demographic variables and surgical factors. Model fixed effects: age, sex, ethnicity, BMI, operative urgency, procedure severity, surgical specialty, number of procedures in the preceding 30 days. Red dot: IMD5, reference level. Blue dots: OR of 30 day mortality for each IMD. Random effect: hospital.

The overall 30-day mortality was 1.3% (*n*=242). In the univariable analysis, patients in the most deprived IMD quintile were 50% more likely to have died at 30 days than those in the least deprived quintile (OR: 1.56, 95% CI: 1.03–2.35; Fig. 4). This association strengthened after adjusting for patient characteristics and surgical factors, with patients in the two most deprived quintiles with significantly greater odds of death (OR IMD2: 1.77, 95% CI: 1.13–2.77; OR IMD1: 1.90, 95% CI: 1.22–2.97). However, in the full multivariable model, although the trend in increased 30-day mortality in more deprived quintiles remained, this association was no longer statistically significant (Supplementary Table S7).

### Sensitivity analyses

Multivariable mixed effects analysis of complete cases yielded ORs for morbidity similar to those of the imputed data analysis, and did not materially alter our findings (Supplementary Table S8). The multivariable analysis was repeated in a cohort of patients <70 yr of age to account for age distribution differences across IMD quintiles (Supplementary Fig. S1). No major discrepancies in our findings were observed compared with those of the full cohort (Supplementary Table S9).

## Discussion

In this large UK national cohort study of inpatient surgery, with a low risk of recruitment bias, we report several key findings. Firstly, patients living in more socioeconomically deprived groups present for surgery at a younger age, are in poorer health, and are more likely to undergo emergency procedures. Secondly, these patients are less likely to undergo cancer surgery or more complex operations. Thirdly, social deprivation is associated with increased risk of morbidity and mortality after surgery. This relationship persists after adjusting for age, sex, ethnicity, and surgical factors, but is no longer statistically significant once preoperative physical status is taken into account. These findings reinforce the complex interplay between socioeconomic deprivation and perioperative risk, and highlight the need for future interventions aimed at improving preoperative physical health in patients from deprived communities to reduce outcome disparities.

The association between social deprivation and short-term morbidity and mortality aligns with findings from previous studies.^4^^,^^7^^,^^8^ For example, Poulton and colleagues^8^ reported an increased risk of 30-day mortality in the most deprived quintile in the emergency laparotomy population, whereas Wan and colleagues^7^ described reduced survival for the most deprived quintile in a mostly elective surgical cohort. To our knowledge, this is the first UK-wide study to examine the impact of social deprivation on outcomes for both elective and emergency surgery across all specialties. Given the non-consenting study design, there is a low risk of recruitment bias, and the sample is likely to be representative of the UK surgical inpatient population. Additionally, this study uses morbidity as a primary outcome, which is an outcome measure affecting a wider population of patients that might be predictive of long-term outcomes.

The variation in patient characteristics across deprivation quintiles correlates well with existing literature.^7^^,^^26^ Patients in the most deprived group present for surgery 10 yr earlier on average than those in less deprived groups. Requiring surgery earlier in life might reflect unhealthy behaviours, limited access to healthcare, or support for management of long-term conditions. This finding is consistent with the higher prevalence of comorbidities previously associated with deprivation, for example, chronic obstructive pulmonary disease and diabetes mellitus,^27^ in the most deprived group. Interestingly, the proportion of patients >80 yr of age was higher in the least deprived group (Supplementary Fig. S1), which might represent a physiologically fitter group of patients selected for surgery, or alternatively reflect survival bias, whereby individuals from less deprived groups are more likely to survive to older age and thus undergo surgery. Future studies could assess the characteristics of patients who would be eligible but did not proceed to surgery for similar conditions, as has been studied in the emergency laparotomy population.^28^^,^^29^

Consistent with previous studies,^7^ those from less deprived backgrounds underwent more complex surgery, and a higher proportion had cancer diagnoses. This finding mirrors National Bowel Cancer Audit reports, which showed fewer than expected diagnoses and major resections among people living in more deprived areas during the pandemic recovery period.^30^^,^^31^ This might reflect reduced uptake of screening or other factors contributing to delayed diagnoses in these communities. In keeping with this hypothesis, patients in more deprived categories were also more likely to present as an emergency. Other studies have shown that patients from deprived backgrounds are more likely to be diagnosed with cancer during emergency presentations.^32^ Late presentation of disease, combined with multimorbidity which can be poorly managed, might reduce the likelihood that people from deprived communities will benefit from high-risk surgery, resulting in nonsurgical or even palliative management.^33^^,^^34^

Understanding the impact and mechanisms by which deprivation is associated with poor surgical outcomes is complex, with many contributing factors. These include increased illness severity at presentation; the effect of pre-existing determinants of health, including lifestyle factors; the overall state of chronic health conditions; limited health literacy and health-seeking behaviour; and variations in quality and access to perioperative care. The intersectionality of ethnicity, disability, and other characteristics related to health inequalities must also be considered.

Our analysis found that the association between deprivation and morbidity and mortality after surgery was independent of measured patient characteristics, including age, sex, and ethnicity, and surgical specialty, urgency, and complexity of surgery. However, after adjusting for comorbidities and markers of preoperative physical fitness, no independent association between deprivation and outcomes remained. This is consistent with findings from several studies,^7^^,^^9^ where deprivation was associated with a greater risk of postoperative complications, but this association was no longer significant after adjusting for comorbidities.

This suggests that patients living in more deprived areas are at risk of worse outcomes after surgery owing to poor overall preoperative health. Contributing factors might include increased severity of comorbid disease or the presenting illness, or untreated modifiable risk factors before surgery. Notably, higher ASA physical status and the presence and severity of anaemia, both strong predictors of outcome in this cohort (Supplementary Table S5), were more frequently observed in the most deprived quintiles. Although social determinants of health, which are not easily addressed on an individual level, might underlie this inequality, the preoperative period could represent an opportunity to reduce health disparities by targeted optimisation of preoperative risk factors, thereby improving outcomes after surgery. In line with NHS England guidance^35^ on early screening and personalised optimisation of risk factors before surgery, individuals living in more deprived areas could be identified as higher risk and benefit from earlier assessment, even for less complex surgery.

Other authors^36^ have raised the question of whether social deprivation, as an independent risk factor, should be incorporated into risk prediction models, to better identify high-risk patients and support preoperative decision-making and planning of postoperative care. The findings of this study do not justify exploring this further, as the increased short-term perioperative risk associated with deprivation is not independent of some variables already included in risk prediction models, for example, ASA physical status. Instead, future work should focus on examining the efficacy of preoperative optimisation interventions that specifically support people from more deprived backgrounds. Investigating the effect of social deprivation on long-term outcomes should also be a priority.

This study has some limitations. A major risk is the potential influence of unmeasured confounding factors on the observed associations. In particular, data on some behavioural factors relevant to deprivation, for example, smoking status, were not collected in the original SNAP-2: EpiCCS study. However, previous research suggests that the association of smoking with surgical outcomes is less strong than that with the conditions resulting from smoking, such as heart and lung disease and cancer. In addition, although widely used, the IMD is a geographic measure and does not measure deprivation on an individual level, raising the possibility of ecological fallacy. However, the relatively small size of LSOAs, the geographic level of IMD, reduces this risk. The composite nature of IMD makes it a valuable tool in this type of research, where relying solely on income or insurance status to measure deprivation can lead to heterogeneity of results. Additionally, the non-consenting design of this study limited our ability to collect individual-level socioeconomic data. This study focused solely on in-hospital major morbidity and mortality; further studies on post-discharge outcomes and readmissions would be valuable. In our cohort, 38% of patients had missing BMI data, likely because of the number of emergency cases where height and weight might not be accurately recorded. Although multiple imputation was used to mitigate the effect of missing data, results related to BMI might not be robust, and although the imputation model included predictors of missingness, there is no absolute certainty that the MAR assumption was met. That said, the complete case analysis did not show any major discrepancies compared with the imputed data. Lastly, it must be acknowledged that SNAP-2: EpiCCS is a study that collected data >8 yr ago. However, with widening health inequalities over the last decade,^37^ understanding and developing strategies to close this gap have never been more pertinent.

In conclusion, although postoperative morbidity and mortality are more common in patients from more deprived postcodes, these differences are not independent of factors relating to preoperative health and fitness. The variations in baseline physical fitness across deprivation quintiles might contribute, suggesting that targeted preoperative optimisation in the most deprived groups could be the single most important intervention to address inequality in surgical outcomes.

## Authors’ contributions

Study concept: SRM

Data preparation: DNJW, RC, EL

Analysis: EL, RC, SPP, BH, SRM

Drafting manuscript: EL

Critical review of the manuscript: all authors

## Funding

London Clinic Hospital (to DW); University College London/University College London Hospitals Surgical Outcomes Research Centre (to DW); University College London Hospitals National Institute for Health Research Biomedical Research Centre and the National Institute for Health Research Central London Patient Safety Research Collaboration (to SRM, SKH); UK Medical Research Council Senior Non-clinical fellowship (ref: MR/Y009398/1 to SPP).

## Declaration of interest

The authors declare that they have no conflicts of interest.
