# Supplementary material for: Social deprivation and morbidity and mortality after surgery: a UK national observational cohort study
Source: Br J Anaesth. 2025 Sep 23;135(5):1193–202. doi: 10.1016/j.bja.2025.07.058 (PMC12597322; doi:10.1016/j.bja.2025.07.058)
Supplement: Multimedia component 1 [file mmc1.docx]

# **Appendix 1: Supplementary Data**

**POMS Criteria**

| **Morbidity Type** | **Criteria** |
| --- | --- |
| Pulmonary | **New requirement for** **oxygen therapy** or **ventilatory support** |
| Infectious | **Currently on antibiotics** or has/had a temperature of >38ºC in the last 24hr |
| Renal | **Presence of oliguria <500ml/24hr**; **increased serum creatinine (>30% from preoperative level);** urinary catheter in situ. |
| Gastrointestinal | Unable to tolerate an enteral diet for any reason including nausea, vomiting, and abdominal distension (use of antiemetic). |
| Cardiovascular | **Diagnostic tests or therapy within the last 24 hr for any of the following: new myocardial infarction or ischemia, hypotension (requiring fluid therapy >200 mL/hr or pharmacological therapy), atrial or ventricular arrhythmias, cardiogenic pulmonary oedema, thrombotic event (requiring anticoagulation).** |
| Neurological | **New focal neurological deficit, confusion, delirium, or coma.** |
| Wound complication | **Wound dehiscence requiring surgical exploration or drainage of pus from the operation wound with or without isolation of organisms.** |
| Haematological | **Requirement for any of the following within the last 24 hr: packed erythrocytes, platelets, fresh-frozen plasma, or cryoprecipitate.** |
| Pain | New postoperative pain significant enough to require parenteral opioids or **regional analgesia** |
| **Supplementary Table 1.** The Post-Operative Morbidity Survery (POMS) Criteria. Those classified as POMS-major criteria are identified in bold. | |

### **Variables included in regression models**

| Variables | | Values |
| --- | --- | --- |
| **Demographic variables** | **Gender** | Male |
|  |  | Female |
|  | **Age, categorised** | 18 to 39 |
|  |  | 40 to 59 |
|  |  | 60 to 69 |
|  |  | 70 to 79 |
|  |  | 80+ |
|  | **Body Mass Index, categorised** | Healthy weight |
|  |  | Underweight |
|  |  | Overweight |
|  |  | Obese |
|  |  | Severely obese |
|  | **Ethnicity** | White |
|  |  | Ethnic minority |
|  | **IMD quintile** | IMD5 – least deprived |
|  |  | IMD4 |
|  |  | IMD3 |
|  |  | IMD2 |
|  |  | IMD1 – most deprived |
| **Surgical factors** | **Procedure Severity**  *(Based on AXA Specialist Procedure Codes)* | Minimal or Intermediate |
|  |  | Major |
|  |  | Extra-major (XMajor) |
|  |  | Complex |
|  | **Operative urgency**  *(Based on NCEPOD classification)* | Elective |
|  |  | Expedited |
|  |  | Urgent |
|  |  | Immediate |
|  | **Surgical specialty** | Orthopaedics |
|  |  | Breast/Endocrine |
|  |  | ENT/MaxFax |
|  |  | Gynaecology/Urology |
|  |  | Gastrointestinal |
|  |  | Neuro/Spine |
|  |  | Thoracic |
|  |  | Vascular |
|  |  | Other |
|  | **Procedures performed in previous 30 days (including current surgery)** | 1 |
|  |  | >1 |
| **Comorbidity and functional status variables** | **ASA-PS** | I+II |
|  |  | III |
|  |  | >=IV |
|  | **Congestive cardiac failure** | Yes |
|  |  | No |
|  | **Stroke/TIA** | Yes |
|  |  | No |
|  | **COPD** | Yes |
|  |  | No |
|  | **Cancer (current or last 5 years)** | Yes |
|  |  | No |
|  | **Coronary artery disease** | Yes |
|  |  | No |
|  | **Diabetes** | None |
|  |  | T1DM |
|  |  | T2DM - Diet Controlled |
|  |  | T2DM - Oral medication |
|  |  | T2DM - Insulin controlled |
|  | **Cardiac history** | None |
|  |  | On cardiac medications |
|  |  | Clinical signs of heart failure |
|  | **Level of dyspnoea pre-operatively**  *(based on NYHA classification)* | None |
|  |  | On exertion |
|  |  | Limiting activities or at rest |
|  | **Anaemia pre-operatively** | No anaemia  *(Hb >130)* |
|  |  | Mild anaemia  *(Hb 110 - 129)* |
|  |  | Moderate anaemia  *(Hb 80-109)* |
|  |  | Severe anaemia  *(Hb <80)* |
| **Supplementary table 2.** Variables included in regression models, subdivided into demographic variables, surgical factors and comorbidity/functional status variables. IMD: Index of Multiple Deprivation, NCEPOD: National Confidential Enquiry into Patient Outcome and Death, ASA-PS: American Society of Anaesthesiologists’ Physical Status Classification, COPD: chronic obstructive pulmonary disease, TIA: Transient Ischaemic Attack, , ENT: Ear, Nose and Throat, MaxFax: Maxillofacial, NYHA: New York Heart Association Functional classification, Hb: Haemoglobin | | |

### **Missing Data and Imputation**

| Variable | Number missing (%) |
| --- | --- |
| **BMI** | 7288 (38.6) |
| **Anaemia pre-operatively** | 1861 (9.9) |
| **IMD quintile** | 1136 (6.0) |
| **Cardiac history** | 81 (0.4) |
| **Level of dyspnoea pre-operatively** | 76 (0.4) |
| **Procedures performed in previous 30 days (including current surgery)** | 65 (0.3) |
| **ASA** | 26 (0.1) |
| **Diabetes** | 18 (0.1) |
| **Ethnicity** | 16 (0.1) |
| **Congestive cardiac failure** | 12 (0.1) |
| **Stroke/TIA** | 12 (0.1) |
| **COPD** | 12 (0.1) |
| **Cancer (current or last 5 years)** | 11 (0.1) |
| **Coronary artery disease** | 9 (0.0) |
| **Operative urgency** | 1 (0.0) |
| **Gender** | 0 (0.0) |
| **Age** | 0 (0.0) |
| **Procedure severity** | 0 (0.0) |
| **Surgical specialty** | 0 (0.0) |
| **Supplementary table 3.** Number and percentage of missing data by predictor variable | |

| Variable | Description | Type |
| --- | --- | --- |
| Gender |  | Binary |
| Age category |  | Categorical |
| Ethnicity | From HES data, collapsed into a binary variable due to small cell sizes | Binary |
| Body Mass Index | Categorised according to standard ranges | Categorical |
| ASA-PS score |  | Categorical |
| IMD quintile | Based on the patient’s usual place of residence, linked with ONS data | Categorical |
| Anaemia pre-operatively | Pre-op haemoglobin, categorised based on WHO classification | Categorical |
| Pre-operative Creatinine* | Transformed to normal distribution, implausible values treated as missing | Continuous |
| Pre-operative Systolic Blood Pressure* | Transformed to normal distribution, implausible values treated as missing | Continuous |
| Day 7 POMS |  | Binary |
| Day 7 POMS major |  | Binary |
| 30-day in hospital mortality | Recorded prospectively by SNAP-2: EpiCCS investigators | Binary |
| 60-day in hospital mortality | Recorded prospectively by SNAP-2: EpiCCS investigators | Binary |
| Coronary Artery Disease |  | Binary |
| Congestive Cardiac Failure |  | Binary |
| Cancer (current or in past 5 years) |  | Binary |
| Stroke/TIA |  | Binary |
| COPD |  | Binary |
| Diabetes | Categorised according to diet, medication or insulin | Categorical |
| Cardiac history | Categorised according to whether patient was taking any cardiac medications, or whether there were clinical signs of heart failure | Categorical |
| Level of dyspnoea pre-operatively | Based on NYHA classification | Categorical |
| Procedure Severity | Based on AXA Specialist Procedure Codes | Categorical |
| Operative Urgency* | Based on NCEPOD Classification | Categorical |
| Surgical Specialty* |  | Categorical |
| Number of procedures performed in the preceding 30 days (including current) |  | Binary |
| Hospital site* | Anonymised code for each hospital | Categorical |
| Supplementary table 4. Independent variables used for multiple imputation. Variables considered to be predictors of missingness are identified with an asterisk. All data was collected prospectively by local SNAP-2: EpiCCS investigators unless otherwise stated. IMD: Index of Multiple Deprivation, POMS: Post-Operative Morbidity Survey, NCEPOD: National Confidential Enquiry into Patient Outcome and Death, ASA-PS: American Society of Anaesthesiologists’ Physical Status Classification, COPD: chronic obstructive pulmonary disease, TIA: Transient Ischaemic Attack, NYHA: New York Heart Association Functional classification, WHO: World Health Organisation | | |

### **Baseline characteristics of cases with and without morbidity**

| **POMS-major Day 7** |  | **Absent** | **Present** | **p** |
| --- | --- | --- | --- | --- |
| Total n (%) |  | 16309 (86.3) | 2592 (13.7) |  |
| Gender | Female | 8973 (55.0) | 1233 (47.6) | <0.001 |
|  | Male | 7336 (45.0) | 1359 (52.4) |  |
| Age | Median (IQR) | 61.0 (45.0 - 73.0) | 71.0 (57.0 - 81.0) | <0.001 |
| Ethnicity | White | 15120 (92.7) | 2448 (94.4) | 0.007 |
|  | Asian | 609 (3.7) | 81 (3.1) |  |
|  | Black | 318 (1.9) | 34 (1.3) |  |
|  | Other | 249 (1.5) | 26 (1.0) |  |
|  | (Missing) | 13 (0.1) | 3 (0.1) |  |
| ASA | I+II | 11694 (71.7) | 831 (32.1) | <0.001 |
|  | III | 4150 (25.4) | 1233 (47.6) |  |
|  | >=IV | 442 (2.7) | 525 (20.3) |  |
|  | (Missing) | 23 (0.1) | 3 (0.1) |  |
| Body Mass Index | Healthy weight | 2465 (15.1) | 317 (12.2) | <0.001 |
|  | Underweight | 211 (1.3) | 57 (2.2) |  |
|  | Overweight | 3893 (23.9) | 381 (14.7) |  |
|  | Obese | 3275 (20.1) | 317 (12.2) |  |
|  | Severely obese | 636 (3.9) | 61 (2.4) |  |
|  | (Missing) | 5829 (35.7) | 1459 (56.3) |  |
| Coronary Artery Disease |  | 1870 (11.5) | 575 (22.2) | <0.001 |
|  | (Missing) | 8 (0.0) | 1 (0.0) |  |
| Congestive Cardiac Failure |  | 448 (2.7) | 216 (8.3) | <0.001 |
|  | (Missing) | 9 (0.1) | 3 (0.1) |  |
| COPD |  | 1232 (7.6) | 382 (14.7) | <0.001 |
|  | (Missing) | 9 (0.1) | 3 (0.1) |  |
| Cancer (Current or last 5 years) |  | 2306 (14.1) | 504 (19.5) | <0.001 |
| Kidney Disease |  | 177 (1.1) | 93 (3.6) | <0.001 |
|  | (Missing) | 10 (0.1) | 5 (0.2) |  |
| Cirrhosis |  | 113 (0.7) | 61 (2.4) | <0.001 |
|  | (Missing) | 9 (0.1) | 4 (0.2) |  |
| Stroke/TIA | Y | 836 (5.1) | 267 (10.3) | <0.001 |
|  | (Missing) | 9 (0.1) | 3 (0.1) |  |
| Level of dyspnoea pre-operatively | None | 13054 (80.0) | 1581 (61.0) | <0.001 |
|  | On exertion | 2259 (13.9) | 500 (19.3) |  |
|  | Limiting activities or at rest | 940 (5.8) | 491 (18.9) |  |
|  | (Missing) | 56 (0.3) | 20 (0.8) |  |
| Cardiac history | None | 13536 (83.0) | 1735 (66.9) | <0.001 |
|  | On cardiac medications | 2409 (14.8) | 685 (26.4) |  |
|  | Signs of heart failure | 295 (1.8) | 160 (6.2) |  |
|  | (Missing) | 69 (0.4) | 12 (0.5) |  |
| Diabetes | None | 14224 (87.2) | 2046 (78.9) | <0.001 |
|  | T1DM | 179 (1.1) | 57 (2.2) |  |
|  | T2DM - Diet Controlled | 423 (2.6) | 104 (4.0) |  |
|  | T2DM - Oral medication | 1077 (6.6) | 240 (9.3) |  |
|  | T2DM - Insulin controlled | 392 (2.4) | 141 (5.4) |  |
|  | (Missing) | 14 (0.1) | 4 (0.2) |  |
| Haemoglobin (g/L) | Median (IQR) | 135.0 (123.0 - 145.0) | 122.0 (105.0 - 136.0) | <0.001 |
| Anaemia pre-operatively | No anaemia | 9128 (56.0) | 926 (35.7) | <0.001 |
|  | Mild anaemia | 4062 (24.9) | 782 (30.2) |  |
|  | Moderate anaemia | 1287 (7.9) | 701 (27.0) |  |
|  | Severe anaemia | 73 (0.4) | 81 (3.1) |  |
|  | (Missing) | 1759 (10.8) | 102 (3.9) |  |
| Creatinine (μmol/L) | Median (IQR) | 74.0 (63.0 - 88.0) | 78.0 (62.0 - 104.0) | <0.001 |
| Procedure Severity | Minimal or Intermediate | 4503 (27.6) | 564 (21.8) | <0.001 |
|  | Major | 5510 (33.8) | 805 (31.1) |  |
|  | Extra-Major | 4060 (24.9) | 622 (24.0) |  |
|  | Complex | 2236 (13.7) | 601 (23.2) |  |
|  | (Missing) | 0 (0.0) | 0 (0.0) |  |
| Operative Urgency | Elective | 9893 (60.7) | 659 (25.4) | <0.001 |
|  | Expedited | 2045 (12.5) | 440 (17.0) |  |
|  | Urgent | 4113 (25.2) | 1270 (49.0) |  |
|  | Immediate | 258 (1.6) | 222 (8.6) |  |
|  | (Missing) | 0 (0.0) | 1 (0.0) |  |
| Procedures performed in previous 30 days (including current surgery) | 1 | 14902 (91.4) | 2061 (79.5) | <0.001 |
|  | >1 | 1353 (8.3) | 520 (20.1) |  |
|  | (Missing) | 54 (0.3) | 11 (0.4) |  |
| Surgical specialty | Ortho | 4976 (30.5) | 925 (35.7) | <0.001 |
|  | Breast/Endocrine | 782 (4.8) | 9 (0.3) |  |
|  | ENT/MaxFax | 801 (4.9) | 61 (2.4) |  |
|  | Gynaecology/Urology | 3665 (22.5) | 161 (6.2) |  |
|  | Gastrointestinal | 3124 (19.2) | 657 (25.3) |  |
|  | Neuro/Spine | 798 (4.9) | 170 (6.6) |  |
|  | Thoracic | 606 (3.7) | 206 (7.9) |  |
|  | Vascular | 404 (2.5) | 122 (4.7) |  |
|  | Other | 1153 (7.1) | 281 (10.8) |  |
|  | (Missing) | 0 (0.0) | 0 (0.0) |  |
| **Supplementary Table 5**. Characteristics of those with morbidity compared to those without on day seven post-op. , POMS: Post-Operative Morbidity Survey, ASA-PS: American Society of Anaesthesiologists’ Physical Status Classification, COPD: chronic obstructive pulmonary disease, TIA: Transient Ischaemic Attack, ENT: Ear, Nose and Throat, MaxFax: Maxillofacial, NYHA: New York Heart Association Functional classification, Hb: Haemoglobin | | | | |

### **Mixed Effects Logistic Regressions**

Calculation of intra-class coefficient

The intra-class coefficient was calculated to estimate how much variation in outcome is dependent on hospital level differences, before adjusting for any other covariates.

For POMS-major: **ICC 0.0605**

*Formula: POMS_major ~ IMD_quintile + (1 | Hospital)*

For 30-day mortality: **ICC 0.0566**

*Formula: 30-day mortality ~ IMD_quintile + (1 | Hospital)*

| **POMS-major** |  | | **Absent** | | **Present** | **Unadjusted OR** | | **Adjusted OR**  **(demographic and surgical factors), mixed effects model** | | **Adjusted OR**  **(full multivariable), mixed effects model** |
| --- | --- | --- | --- | --- | --- | --- | --- | --- | --- | --- |
| IMD quintile | IMD5 – least deprived | | 3170 (87.6) | | 448 (12.4) | ref | | ref | | ref |
|  | IMD4 | | 3068 (86.0) | | 499 (14.0) | 1.15 (1.00-1.32, p=0.046) | | 1.12 (0.97-1.31, p=0.129) | | 1.06 (0.91-1.24, p=0.464) |
|  | IMD3 | | 2954 (86.7) | | 454 (13.3) | 1.09 (0.95-1.25, p=0.242) | | 1.11 (0.95-1.29, p=0.210) | | 1.03 (0.88-1.21, p=0.707) |
|  | IMD2 | | 3008 (86.0) | | 489 (14.0) | 1.15 (1.01-1.32, p=0.040) | | 1.30 (1.11-1.52, p=0.001) | | 1.14 (0.97-1.34, p=0.109) |
|  | IMD1 – most deprived | | 3153 (85.8) | | 522 (14.2) | 1.17 (1.03-1.34, p=0.018) | | 1.35 (1.16-1.58, p<0.001) | | 1.12 (0.95-1.32, p=0.172) |
| Gender | Female | | 8973 (87.9) | | 1233 (12.1) | ref | | ref | | ref |
|  | Male | | 7336 (84.3) | | 1359 (15.6) | 1.35 (1.24-1.46, p<0.001) | | 1.21 (1.10-1.33, p<0.001) | | 1.28 (1.16-1.42, p<0.001) |
| Age Category | 18 to 39 | | 3153 (94.8) | | 209 (6.2) | ref | | ref | | ref |
|  | 40 to 59 | | 4600 (89.9) | | 515 (10.1) | 1.69 (1.43-2.00, p<0.001) | | 2.20 (1.84-2.64, p<0.001) | | 1.64 (1.36-1.99, p<0.001) |
|  | 60 to 69 | | 3331 (87.7) | | 468 (12.3) | 2.12 (1.79-2.51, p<0.001) | | 3.02 (2.51-3.64, p<0.001) | | 1.84 (1.51-2.25, p<0.001) \| |
|  | 70 to 79 | | 3296 (82.7) | | 689 (17.3) | 3.15 (2.68-3.71, p<0.001) | | 4.65 (3.88-5.57, p<0.001) | | 2.41 (1.97-2.94, p<0.001) |
|  | 80+ | | 1929 (73.1) | | 711 (26.9) | 5.56 (4.72-6.55, p<0.001) | | 6.85 (5.69-8.25, p<0.001) | | 2.91 (2.36-3.59, p<0.001) |
| Ethnicity | White | | 15120 (86.1) | | 2448 (13.9) | ref | | ref | | ref |
|  | Ethnic minority | | 1176 (89.3) | | 141 (10.7) | 0.74 (0.62-0.89, p=0.001) | | 0.92 (0.75-1.13, p=0.431) | | 0.87 (0.70-1.08, p=0.208) |
| Body Mass Index | Healthy weight | | 2465 (88.6) | | 317 (11.4) | ref | | ref | | ref |
|  | Underweight | | 211 (78.7) | | 57 (21.3) | 2.13 (1.71-2.64, p<0.001) | | 1.46 (1.14-1.89, p=0.003) | | 1.09 (0.83-1.43, p=0.538) |
|  | Overweight | | 3893 (91.1) | | 381 (8.9) | 0.70 (0.61-0.80, p<0.001) | | 0.79 (0.68-0.92, p=0.003) | | 0.88 (0.75-1.04, p=0.136) |
|  | Obese | | 3275 (91.2) | | 317 (8.8) | 0.70 (0.62-0.80, p<0.001) | | 0.90 (0.77-1.04, p=0.162) | | 0.92 (0.78-1.08, p=0.308) |
|  | Severely obese | | 636 (91.2) | | 61 (8.8) | 0.72 (0.57-0.91, p=0.006) | | 1.02 (0.78-1.33, p=0.891) | | 0.72 (0.54-0.97, p=0.028) |
| Coronary Artery Disease |  | | 1870 (76.5) | | 575 (23.5) | 2.20 (1.98-2.44, p<0.001) | | - | | 0.83 (0.72-0.96, p=0.011) |
| Congestive Cardiac Failure |  | | 448 (67.5) | | 216 (32.5) | 3.23 (2.73-3.82, p<0.001) | | - | | 0.88 (0.71-1.10, p=0.255) |
| COPD |  | | 1232 (76.3) | | 382 (23.7) | 2.12 (1.87-2.39, p<0.001) | | - | | 1.10 (0.95-1.29, p=0.210) |
| Cancer (Current or last 5 years) |  | | 2306 (82.1) | | 504 (17.9) | 1.47 (1.32-1.63, p<0.001) | | - | | 1.12 (0.98-1.28, p=0.107) |
| Stroke/TIA | Y | | 836 (75.8) | | 267 (24.2) | 2.13 (1.84-2.46, p<0.001) | | - | | 1.05 (0.89-1.25, p=0.547) |
| Level of dyspnoea pre-operatively | None | | 13054 (89.2) | | 1581 (10.8) | ref | | - | | ref |
|  | On exertion | | 2259 (81.9) | | 500 (18.1) | 1.83 (1.64-2.04, p<0.001) | | - | | 1.01 (0.88-1.17, p=0.863) |
|  | Limiting activities or at rest | | 940 (65.7) | | 491 (34.3) | 4.31 (3.82-4.87, p<0.001) | | - | | 1.36 (1.15-1.61, p<0.001) |
| Cardiac history | None | | 13536 (88.6) | | 1735 (11.4) | ref | | - | | ref |
|  | On cardiac medications | | 2409 (77.9) | | 685 (22.1) | 2.22 (2.01-2.45, p<0.001) | | - | | 0.99 (0.86-1.14, p=0.920) |
|  | Signs of heart failure | | 295 (64.8) | | 160 (35.2) | 4.24 (3.47-5.17, p<0.001) | | - | | 1.58 (1.23-2.02, p<0.001) |
| Diabetes | None | | 14224 (87.4) | | 2046 (12.6) | ref | | - | | ref |
|  | T1DM | | 179 (75.8) | | 57 (24,2) | 2.21 (1.63-2.99, p<0.001) | | - | | 1.46 (1.03-2.07, p=0.033) |
|  | T2DM - Diet Controlled | | 423 (80.2) | | 104 (19.7) | 1.71 (1.37-2.13, p<0.001) | | - | | 1.14 (0.88-1.47, p=0.324) |
|  | T2DM - Oral medication | | 1077 (81.8) | | 240 (18.2) | 1.55 (1.34-1.79, p<0.001) | | - | | 1.04 (0.88-1.25, p=0.639) |
|  | T2DM - Insulin controlled | | 392 (73.5) | | 141 (26.5) | 2.50 (2.05-3.04, p<0.001) | | - | | 1.29 (1.01-1.63, p=0.038) |
| Anaemia pre-operatively | No anaemia | | 9128 (90.8) | | 926 (9.2) | ref | | - | | ref |
|  | Mild anaemia | | 4062 (83.9) | | 782 (16.1) | 1.94 (1.75-2.15, p<0.001) | | - | | 1.45 (1.28-1.63, p<0.001) |
|  | Moderate anaemia | | 1287 (64.7) | | 701 (35.3) | 5.59 (4.99-6.26, p<0.001) | | - | | 2.43 (2.11-2.80, p<0.001) |
|  | Severe anaemia | | 73 (47.4) | | 81 (52.6) | 10.38 (7.56-14.24, p<0.001) | | - | | 3.22 (2.20-4.71, p<0.001) |
| ASA | I+II | | 11694 (93.4) | | 831 (6.6) | ref | | - | | ref |
|  | III | | 4150 (77.1) | | 1233 (22.9) | 4.18 (3.80-4.60, p<0.001) | | - | | 2.20 (1.94-2.49, p<0.001) |
|  | >=IV | | 442 (45.7) | | 525 (54.3) | 16.73 (14.47-19.33, p<0.001) | | - | | 4.52 (3.72-5.50, p<0.001) |
| Procedure Severity | Minimal or Intermediate | | 4503 (88.9) | | 564 (11.1) | ref | | ref | | ref |
|  | Major | | 5510 (87.3) | | 805 (12.7) | 1.17 (1.04-1.31, p=0.008) | | 1.39 (1.22-1.60, p<0.001) | | 1.41 (1.22-1.63, p<0.001) |
|  | Extra - Major | | 4060 (86.7) | | 622 (13.3) | 1.22 (1.08-1.38, p=0.001) | | 1.46 (1.25-1.70, p<0.001) | | 1.49 (1.27-1.74, p<0.001) |
|  | Complex | | 2236 (78.8) | | 601 (21.2) | 2.15 (1.89-2.43, p<0.001) | | 2.37 (2.01-2.80, p<0.001) | | 2.42 (2.04-2.89, p<0.001) |
| Operative Urgency | Elective | | 9893 (93.8) | | 659 (6.2) | ref | | ref | | ref |
|  | Expedited | | 2045 (82.8) | | 440 (17.7) | 3.23 (2.84-3.68, p<0.001) | | 2.89 (2.51-3.33, p<0.001) | | 2.23 (1.92-2.59, p<0.001) |
|  | Urgent | | 4113 (76.4) | | 1270 (23.6) | 4.64 (4.19-5.13, p<0.001) | | 4.52 (4.01-5.09, p<0.001) | | 3.29 (2.91-3.72, p<0.001) |
|  | Immediate | | 258 (53.8) | | 222 (46.2) | 12.91 (10.62-15.71, p<0.001) | | 14.10 (11.18-17.79, p<0.001) | | 7.60 (5.91-9.77, p<0.001) |
| Procedures performed in previous 30 days (including current surgery) | 1 | | 14902 (87.9) | | 2061 (12.1) | ref | | ref | | ref |
|  | >1 | | 1353 (72.2) | | 520 (27.8) | 2.78 (2.49-3.11, p<0.001) | | 2.17 (1.91-2.46, p<0.001) | | 1.72 (1.50-1.98, p<0.001) |
| Surgical specialty | Ortho | | 4976 (84.3) | | 925 (15.7) | ref | | ref | | ref |
|  | Breast/Endocrine | | 782 (98.9) | | 9 (1.1) | 0.06 (0.03-0.12, p<0.001) | | 0.13 (0.07-0.26, p<0.001) | | 0.16 (0.08-0.30, p<0.001) |
|  | ENT/MaxFax | | 801 (92.9) | | 61 (7.1) | 0.41 (0.31-0.54, p<0.001) | | 0.81 (0.60-1.10, p=0.165) | | 0.72 (0.52-0.99, p=0.041) |
|  | Gynaecology/Urology | | 3665 (95.8) | | 161 (4.2) | 0.24 (0.20-0.28, p<0.001) | | 0.42 (0.35-0.51, p<0.001) | | 0.43 (0.35-0.52, p<0.001) |
|  | Gastrointestinal | | 3124 (82.6) | | 657 (17.4) | 1.13 (1.01-1.26, p=0.027) | | 1.35 (1.19-1.54, p<0.001) | | 1.39 (1.21-1.59, p<0.001) |
|  | Neuro/Spine | | 798 (82.4) | | 170 (17.6) | 1.15 (0.96-1.37, p=0.137) | | 1.17 (0.94-1.54, p=0.161) | | 1.22 (0.96-1.52, p=0.098) |
|  | Thoracic | | 606 (74.6) | | 206 (25.4) | 1.83 (1.54-2.17, p<0.001) | | 1.60 (1.28-2.00, p<0.001) | | 1.15 (0.91-1.47, p=0.239) |
|  | Vascular | | 404 (76.8) | | 122 (23.2) | 1.62 (1.31-2.01, p<0.001) | | 0.85 (0.65-1.10, p=0.218) | | 0.64 (0.48-0.83, p=0.001) |
|  | Other | | 1153 (80.4) | | 281 (19.6) | 1.31 (1.13-1.52, p<0.001) | | 1.32 (1.10-1.59, p=0.002) | | 1.10 (0.90-1.34, p=0.343) |
| **Supplementary Table 6a.** Univariable and multivariable mixed effects analysis of POMS-major morbidity and IMD quintiles (least-deprived group IMD5 as reference). Model covariates: age, gender, ethnicity, BMI, surgical factors (+ ASA, comorbid disease, pre-operative anaemia - full multivariable). POMS: Post-operative morbidity survey, OR: Odds Ratio, IMD: Index of Multiple Deprivation, COPD: chronic obstructive pulmonary disease, TIA: Transient Ischaemic Attack, ASA: American Society of Anaesthesiologists’ Physical Status Classification, ENT: Ear, Nose and Throat, MaxFax: Maxillofacial | | | | | | | | | | |
| **POMS-major** | | **Model fit Statistics** | | **Unadjusted single level model** | | | **Adjusted mixed effects model**  **(demographic and surgical factors)** | | **Adjusted mixed effects**  **(full multivariable)** | |
|  |  | Log-Likelihood | | -7551.774 | | | -6122.523 | | -5762.078 | |
|  |  | AIC | | 15113.55 | | | 12307.05 | | 11550.16 | |
|  |  | BIC | | 15152.78 | | | 12550.3 | | 11934.66 | |
|  |  | ICC | | - | | | 0.037 | | 0.038 | |
|  | | Deviance | | 15103.55 | | | 11991.58 | | 11204.88 | |
| **Supplementary Table 6b**. Model fit statistics for models for the POMS-major mortality outcome. AIC: Akaike Information Criterion, BIC: Bayesian Information Criterion, ICC: Intraclass Correlation Coefficient | | | | | | | | | | |

| **30-day mortality** |  | **Absent** | **Present** | **Unadjusted OR** | **Adjusted OR**  **(demographic and surgical factors)** | **Adjusted OR**  **(full multivariable)** |
| --- | --- | --- | --- | --- | --- | --- |
| IMD quintile | IMD5 – least deprived | 3580 (98.9) | 38 (1.1) | ref | ref | ref |
|  | IMD4 | 3521 (98.7) | 46 (1.3) | 1.24 (0.80-1.91, p=0.333) | 1.26 (0.79-1.98, p=0.329) | 1.20 (0.75-1.93, p=0.445) |
|  | IMD3 | 3364 (98.7) | 44 (1.3) | 1.23 (0.79-1.91, p=0.356) | 1.21 (0.76-1.93, p=0.414) | 1.18 (0.73-1.91, p=0.506) |
|  | IMD2 | 3444 (98.5) | 53 (1.5) | 1.44 (0.95-2.19, p=0.087) | 1.77 (1.13-2.77, p=0.013) | 1.38 (0.86-2.21, p=0.176) |
|  | IMD1 – most deprived | 3616 (98.4) | 59 (1.6) | 1.56 (1.03-2.35, p=0.034) | 1.90 (1.22-2.97, p=0.005) | 1.55 (0.98-2.45, p=0.063) |
| Gender | Female | 10104 (99.0) | 102 (1.0) | ref | ref | ref |
|  | Male | 8538 (98.2) | 157 (1.8) | 1.82 (1.42-2.34, p<0.001) | 1.68 (1.28-2.21, p<0.001) | 1.65 (1.24-2.20, p=0.001) |
| Age Category | 18 to 39 | 3350 (99.6) | 12 (0.4) | ref | ref | ref |
|  | 40 to 59 | 5084 (99.4) | 31 (0.6) | 1.70 (0.87-3.32, p=0.118) | 2.39 (1.21-4.71, p=0.012) | 1.18 (0.58-2.42, p=0.651) |
|  | 60 to 69 | 3763 (99.1) | 36 (0.9) | 2.67 (1.39-5.14, p=0.003) | 4.48 (2.28-8.80, p<0.001) | 1.55 (0.75-3.21, p=0.239) |
|  | 70 to 79 | 3904 (98.0) | 81 (2.0) | 5.79 (3.15-10.64, p<0.001) | 9.87 (5.24-18.57, p<0.001) | 2.70 (1.35-5.42, p=0.005) |
|  | 80+ | 2541 (96.3) | 99 (3.7) | 10.88 (5.96-19.85, p<0.001) | 15.08 (8.00-28.42, p<0.001) | 3.34 (1.65-6.79, p=0.001) |
| Ethnicity | White | 17323 (98.6) | 245 (1.4) | ref | ref | ref |
|  | Ethnic minority | 1303 (98.9) | 14 (1.1) | 0.76 (0.44-1.30, p=0.317) | 1.13 (0.63-2.02, p=0.677) | 1.06 (0.58-1.94, p=0.854) |
| Body Mass Index | Healthy weight | 2750 (98.9) | 32 (1.1) | ref | ref | ref |
|  | Underweight | 261 (97.4) | 7 (2.6) | 2.16 (1.33-3.51, p=0.002) | 1.21 (0.70-2.08, p=0.497) | 0.88 (0.50-1.54, p=0.643) |
|  | Overweight | 4253 (99.5) | 21 (0.5) | 0.45 (0.31-0.65, p<0.001) | 0.54 (0.36-0.81, p=0.003) | 0.58 (0.38-0.90, p=0.016) |
|  | Obese | 3577 (99.6) | 15 (0.4) | 0.38 (0.25-0.58, p<0.001) | 0.55 (0.35-0.87, p=0.010) | 0.51 (0.31-0.84, p=0.008) |
|  | Severely obese | 694 (99.6) | 3 (0.4) | 0.31 (0.12-0.84, p=0.021) | 0.54 (0.19-1.48, p=0.229) | 0.30 (0.10-0.89, p=0.030) |
| Coronary Artery Disease |  | 2357 (96.4) | 88 (3.6) | 3.56 (2.74-4.62, p<0.001) | - | 0.97 (0.69-1.35, p=0.845) |
| Congestive Cardiac Failure |  | 620 (93.4) | 44 (6.6) | 6.04 (4.32-8.43, p<0.001) | - | 1.09 (0.71-1.68, p=0.680) |
| COPD |  | 1573 (97.5) | 41 (2.5) | 2.05 (1.46-2.87, p<0.001) | - | 0.69 (0.47-1.02, p=0.062) |
| Cancer (Current or last 5 years) |  | 2758 (98.1) | 52 (1.9) | 1.45 (1.07-1.98, p=0.017) | - | 0.97 (0.68-1.38, p=0.852) |
| Stroke/TIA |  | 1066 (96.6) | 37 (3.4) | 2.75 (1.93-3.92, p<0.001) | - | 1.06 (0.71-1.58, p=0.792) |
| Level of dyspnoea pre-operatively | None | 14514 (99.2) | 121 (0.8) | ref | - | ref |
|  | On exertion | 2716 (98.4) | 43 (1.6) | 1.91 (1.34-2.70, p<0.001) | - | 0.93 (0.62-1.39, p=0.725) |
|  | Limiting activities or at rest | 1340 (93.6) | 91 (6.4) | 8.23 (6.24-10.86, p<0.001) | - | 1.88 (1.30-2.72, p=0.001) |
| Cardiac history | None | 15138 (99.1) | 133 (0.9) | ref | - | ref |
|  | On cardiac medications | 2995 (96.8) | 99 (3.2) | 3.80 (2.92-4.94, p<0.001) | - | 1.16 (0.82-1.66, p=0.403) |
|  | Signs of heart failure | 430 (94.5) | 25 (5.5) | 6.83 (4.42-10.54, p<0.001) | - | 1.51 (0.90-2.57, p=0.119) |
| Diabetes | None | 16088 (98.9) | 182 (1.1) | ref | - | ref |
|  | T1DM | 229 (97.0) | 7 (3.0) | 2.70 (1.26-5.81, p=0.011) | - | 1.92 (0.83-4.44, p=0.125) |
|  | T2DM - Diet Controlled | 511 (97.0) | 16 (3.0) | 2.77 (1.65-4.65, p<0.001) | - | 1.78 (0.99-3.21, p=0.055) |
|  | T2DM - Oral medication | 1278 (97.0) | 39 (3.0) | 2.70 (1.90-3.83, p<0.001) | - | 1.89 (1.24-2.88, p=0.003) |
|  | T2DM - Insulin controlled | 518 (97.2) | 15 (2.9) | 2.56 (1.50-4.37, p=0.001) | - | 1.13 (0.62-2.06, p=0.698) |
| Anaemia pre-operatively | No anaemia | 9992 (99.4) | 62 (0.6) | ref | - | ref |
|  | Mild anaemia | 4769 (98.5) | 75 (1.5) | 2.57 (1.84-3.59, p<0.001) | - | 1.47 (1.02-2.13, p=0.040) |
|  | Moderate anaemia | 1896 (95.4) | 92 (4.6) | 8.31 (6.02-11.47, p<0.001) | - | 2.04 (1.39-2.98, p<0.001) |
|  | Severe anaemia | 140 (90.9) | 14 (9.1) | 17.44 (9.76-31.14, p<0.001) | - | 2.24 (1.12-4.48, p=0.022) |
| ASA | I+II | 12503 (99.8) | 22 (0.2) | ref | - | ref |
|  | III | 5278 (98.0) | 105 (2.0) | 11.30 (7.13-17.92, p<0.001) | - | 4.48 (2.66-7.52, p<0.001) |
|  | >=IV | 835 (86.3) | 132 (13.7) | 89.76 (56.85-141.73, p<0.001) \| | - | 13.85 (7.93-24.21, p<0.001) |
| Procedure Severity | Minimal or Intermediate | 5007 (98.8) | 60 (1.2) | ref | ref | ref |
|  | Major | 6227 (98.6) | 88 (1.4) | 1.18 (0.85-1.64, p=0.328) | 1.27 (0.86-1.87, p=0.227) | 1.23 (0.82-1.87, p=0.318) |
|  | Extra- XMajor | 4629 (98.9) | 53 (1.1) | 0.96 (0.66-1.39, p=0.810) | 1.12 (0.72-1.74, p=0.621) | 1.06 (0.66-1.69, p=0.812) |
|  | Complex | 2779 (98.0) | 58 (2.0) | 1.74 (1.21-2.51, p=0.003) | 1.40 (0.88-2.24, p=0.157) | 1.30 (0.80-2.12, p=0.290) |
| Operative Urgency | Elective | 10523 (99.7) | 29 (0.3) | ref | ref | ref |
|  | Expedited | 2452 (98.7) | 33 (1.3) | 4.88 (2.96-8.06, p<0.001) | 3.76 (2.25-6.28, p<0.001) | 2.36 (1.39-3.99, p=0.001) |
|  | Urgent | 5251 (97.5) | 132 (2.5) | 9.12 (6.09-13.65, p<0.001) | 7.21 (4.69-11.07, p<0.001) | 3.71 (2.37-5.80, p<0.001) |
|  | Immediate | 415 (86.5) | 65 (13.5) | 56.83 (36.29-89.00, p<0.001) | 40.87 (24.90-67.07, p<0.001) | 12.99 (7.62-22.16, p<0.001) |
| Procedures performed in previous 30 days (including current surgery) | 1 | 16755 (98.8) | 208 (1.2) | ref | ref | ref |
|  | >1 | 1824 (97.4) | 49 (2.6) | 2.19 (1.60-3.01, p<0.001) | 1.30 (0.93-1.83, p=0.130) | 0.93 (0.64-1.33, p=0.679) |
| Surgical specialty | Ortho | 5826 (98.7) | 75 (1.3) | ref | ref | ref |
|  | Breast/Endocrine | 790 (99.9) | 1 (0.1) | 0.10 (0.01-0.71, p=0.021) | 0.40 (0.05-2.95, p=0.369) | 0.67 (0.09-5.05, p=0.700) |
|  | ENT/MaxFax | 853 (99.0) | 9 (1.0) | 0.82 (0.41-1.64, p=0.575) | 1.79 (0.82-3.92, p=0.142) | 1.67 (0.71-3.946 p=0.241) |
|  | Gynaecology/Urology | 3815 (99.7) | 11 (0.3) | 0.22 (0.12-0.42, p<0.001) | 0.52 (0.26-1.02, p=0.057) | 0.61 (0.30-1.24, p=0.171) |
|  | Gastrointestinal | 3714 (98.2) | 67 (1.8) | 1.40 (1.01-1.95, p=0.046) | 1.58 (1.10-2.29, p=0.014) | 1.84 (1.23-2.73, p=0.003) |
|  | Neuro/Spine | 951 (98.3) | 16 (1.7) | 1.39 (0.82-2.36, p=0.226) | 1.61 (0.88-2.96, p=0.123) | 1.81 (0.95-3.44, p=0.070) |
|  | Thoracic | 796 (98.0) | 16 (2.0) | 1.56 (0.91-2.69, p=0.109) | 1.75 (0.93-3.33, p=0.083) | 0.98 (0.49-1.95, p=0.953) |
|  | Vascular | 497 (94.5) | 29 (5.5) | 4.53 (2.92-7.03, p<0.001) | 2.22 (1.26-3.89, p=0.005) | 1.89 (1.05-3.37, p=0.033) |
|  | Other | 1400 (97.6) | 35 (2.4) | 1.89 (1.25-2.84, p=0.002) | 1.72 (1.04-2.85, p=0.034) | 1.42 (0.83-2.42, p=0.206) |
| **Supplementary Table 7a.** Univariable and multivariable mixed effects analysis of 30-day mortality and IMD quintiles (least-deprived group IMD5 as reference). Model covariates: age, gender, ethnicity, BMI, surgical factors (+ ASA, comorbid disease, pre-operative anaemia - full multivariable). POMS: Post-operative morbidity survey, OR: Odds Ratio, IMD: Index of Multiple Deprivation, COPD: chronic obstructive pulmonary disease, TIA: Transient Ischaemic Attack, ASA: American Society of Anaesthesiologists’ Physical Status Classification, ENT: Ear, Nose and Throat, MaxFax: Maxillofacial | | | | | | |

| **30-day mortality** | **Model fit Statistics** | **Unadjusted single level model** | **Adjusted mixed effects model**  **(demographic and surgical factors)** | **Adjusted mixed effects**  **(full multivariable)** |
| --- | --- | --- | --- | --- |
|  | Log-Likelihood | -1364.888 | -1047.572 | -914.093 |
|  | AIC | 2739.776 | 2157.144 | 1926.186 |
|  | BIC | 2779.011 | 2400.401 | 2310.688 |
|  | ICC | - | 0.025 | 0.009 |
|  | Deviance | 2729.776 | 2059.883 | 1816.489 |
| **Supplementary Table 7b**. Model fit statistics for models for the 30-day mortality outcome. AIC: Akaike Information Criterion, BIC: Bayesian Information Criterion, ICC: Intraclass Correlation Coefficient | | | | |

### **Complete Case Analysis**

| **POMS-major** | | **Unadjusted OR** | **Adjusted for surgical and demographic factors** | **Full multivariable)** |
| --- | --- | --- | --- | --- |
| **Number of observations (n)** | | **17765** | **10957** | **9962** |
| IMD quintile | IMD5 – least deprived | ref | ref | ref |
|  | IMD4 | 1.15 (1.00-1.32, p = 0.044) | 1.06 (0.92-1.42, p =0.217) | 1.07 (0.85– 1.34, p = 0.570) |
|  | IMD3 | 1.09 (0.95-1.25, p = 0.240) | 1.04 (0.83 – 1.30, p = 0.732) | 0.92 (0.72 – 1.17, p = 0.481) |
|  | IMD2 | 1.15 (1.00-1.32, p = 0.046) | 1.28 (1.03-1.60, p = 0.029) | 1.09 (0.86 – 1.38, p = 0.450) |
|  | IMD1 – most deprived | 1.17 (1.02 – 1.34, p = 0.022) | 1.36 (1.09 – 1.71, p = 0.006) | 1.12 (0.88 – 1.42, p = 0.347) |
| **Supplementary Table 8**. Complete case analysis: unadjusted and mixed effects multivariable analysis of POMS-major and IMD quintiles (least-deprived group IMD5 as reference), using hospital site as a random intercept. Model covariates: age, gender, ethnicity, BMI, surgical factors, ASA, comorbid disease, pre-operative anaemia. POMS: Post-operative morbidity survey, OR: Odds Ratio, IMD: Index of Multiple Deprivation, ASA: American Society of Anaesthesiologists’ Physical Status Classification. | | | | |

NB In the complete case analysis of the effect of IMD on the secondary outcome of 30-day mortality, the mixed-effects full multivariable model did not converge. This was likely due to the small number of patients who died within 30 days (n = 69) and the small number of complete cases (n =9962). The single-level model (without hospital as a random effect) did converge and showed no significant difference in ORs compared to the main analysis.

### **Age differences across data set**


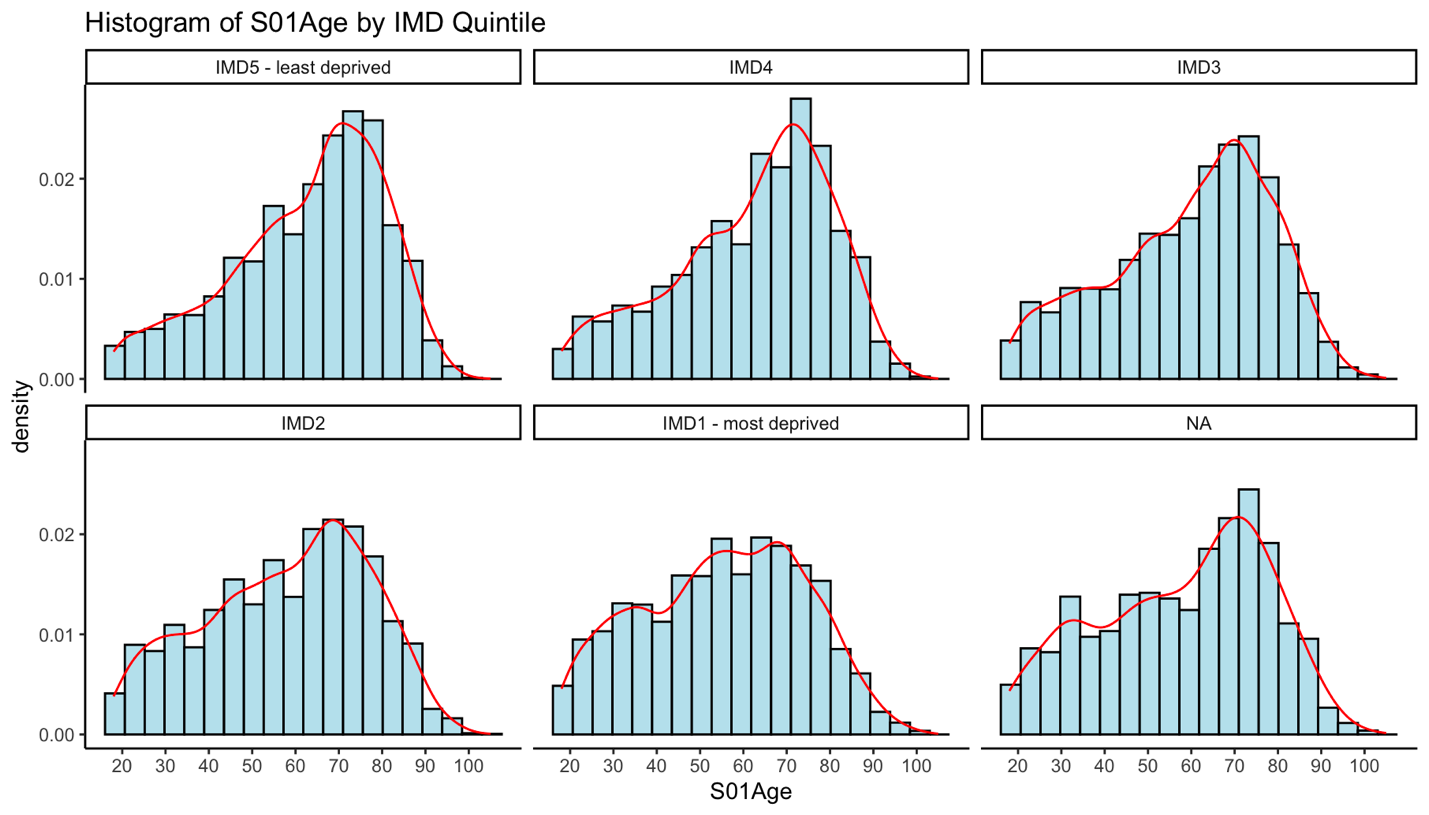


**Supplementary Fig.1** : Histograms showing distribution of age by IMD quintile

| **Outcomes in those <70 years** |  | **POMS-major (unadjusted OR)** | **POMS-major (Full multivariable)** |
| --- | --- | --- | --- |
| IMD quintile | IMD5 – least deprived | ref | ref |
|  | IMD4 | 1.06 (0.86-1.32, p = 0.57) | 0.97 (0.76-1.24, p =0.80) |
|  | IMD3 | 1.15 (0.94-1.42, p = 0.18) | 1.06 (0.83 – 1.35, p = 0.63) |
|  | IMD2 | 1.20 (0.98-1.47, p = 0.07) | 1.09 (0.86-1.38, p = 0.45) |
|  | IMD1 – most deprived | 1.38 (1.14 – 1.67, p = 0.001) | 1.17 (0.93– 1.47, p = 0.17) |
| **Supplementary Table 9**. Mixed effects multivariable analysis of POMS-major and IMD, in those <70 years. For Model covariates: age, gender, ethnicity, BMI, surgical factors, ASA, comorbid disease, pre-operative anaemia. POMS: Post-operative morbidity survey, OR: Odds Ratio, IMD: Index of Multiple Deprivation, ASA: American Society of Anaesthesiologists’ Physical Status Classification. | | | |

NB In the <70 years sample, the mixed-effects full multivariable model for the 30-day mortality outcome did not converge. This is likely due to the small number of patients who died within 30 days in the sample. The single-level model (without hospital as a random effect) did converge and showed no major discrepancy between ORs compared to the main analysis.

# **Appendix 2:** List of SNAP-2 Collaborators

Mrs Kathy Malinovszky

Dr Shilpa Rawat

Dr Samuel Tyrrell

Dr Janakan Anandarajah

Dr Nicola Ball

Dr Catherine Chapman

Dr Amanda Ebejer

Dr Maire Gallagher

Dr Sarah Goff

Dr Rebecca Jackson

Dr Kathryn James

Dr Claire Jones

Mr Hari Nageswaran

Dr Harriet Pudge

Dr Thomas Sheppard

Dr Owen Vale

Dr Catrin Williams

Ms Leanne Quinn

Dr Sonia Sathe

Dr Tom Williams

Dr Lewys Winfield-Young

Dr Lalindra Bandara

Dr Dennis Barnes

Dr Alison Campbell

Mrs Lynda Connor

Mrs Amanda Cook

Miss Samantha Evans

Dr Irina Halfacree

Mrs Rachel Harford

Mrs Catherine Harris

Mrs Sharon Jones

Ms Serah Mungai

Dr Anand Perumal

Mrs Trudy Smith

Dr James Spargo

Ms Sharon Storton

Mr Caradog Thomas

Dr Charlotte Thomas

Miss Marie Williams

Mrs Helen Worrell

Dr Chandini Chuni

Dr Jasna Comara

Dr Brian Conway

Dr Zara Eagle

Dr Greg Foster

Dr Dermot Moloney

Dr Chris Platt

Dr Alison Thorne

Ms Lisa Armstrong

Dr Albert Brennan

Dr Justine Burns

Dr Chantal Busby

Ms Mechele Couch-Upite

Ms Emma Dooks

Dr Sally Dunlop

Dr Anya Eijk

Dr Ramana Govindaraju

Dr Tamsin Gregory

Dr Alka Grover

Ms Brigid Hairsine

Dr Sarah Hennell

Ms Philippa Hill

Dr Thomas Hollins

Dr Julian Hood

Ms Lisa Horner

Ms Helen Hothersall

Dr Rachel Ingham

Dr Richard Jackson

Ms Amy Kitching

Ms Claire Kurasz

Dr Wendy Lum Hee

Ms Sarah Munsie

Dr Ildiko Nemeth

Ms Carole Paley

Dr Prabhakaran Premraj

Ms Sue Redhead

Dr John Scriven

Ms Alison Shaw

Ms Liz Shenton

Dr Hemantha Shiva

Ms Josie Snell

Ms Lucy Sootheran

Dr Josephine Stewart

Dr Frank Swinton

Dr Claire Totten

Dr Liz Varghese

Dr Sarah Vest

Dr Ben Wetherell

Dr Timothy Alce

Dr Phillippa Falkner

Dr Vincent Hamlyn

Dr Saima Hashmi

Mrs Angie Organ

Dr Frances Taylor

Dr Maria Tritean

Dr Richard Wassall

Dr Jade Woolley

Dr Poonam Bopanna

Dr Kevin Draper

Dr Lucy Emmett

Dr Ellie Fisher

Dr Cat Griffiths

Ms Una Gunter

Dr Rebecca Harris

Dr Helen Jewitt

Dr Sonal Lodhi

Dr Elana Owen

Dr James Tozer

Dr Anja Kuttler

Dr Christina Lalani

Ms Beth Peers

Ms Hayley Tarft

Mrs Claire Atkinson

Miss Maria Croft

Mrs Victoria Frost

Dr Richard George

Ms Catherine Gray

Dr Mark MacGregor

Mrs Lisa Sharpe

Dr Christopher Skeoch

Dr Emma Tyson

Dr Martha Wrigley

Dr Tahir Abbas

Dr Niraj Barot

Dr Steve Cole

Dr Ritesh Ganesh

Dr Christopher Groves

Dr Lindsey Iles

Dr Hristina Petkova

Dr Shabir Qadri

Dr Clovis Rau

Dr Jack Roberts

Dr Nadeem Shakir

Dr Julia Brown

Dr Sunil Chaurasia

Ms Mishell Cunningham

Ms Allison Daniels

Dr Emily Gannon

Dr Annette Haines

Dr Nicola Johnson

Mr Paul Ogle

Dr Srinivasan Perumal

Ms Lisa Zeidan

Dr Laura Ashton

Dr Emma Butterfield

Dr Charlotte Marriot

Dr Otto Ernst Mohr

Dr Michael Shaw

Dr Christopher Smith

Dr Hew David Torrance

Dr Farkhunda Waqas

Dr Sibtain Anwar

Dr Thomas Garth

Dr Liam Gleeson

Dr Cormac O'Connor

Dr Kimberley Plummer

Dr Gerhardus Van Rensberg

Dr Peter Wicks

Dr Tom Abbott

Dr Katherine Brown

Dr Jon Fenn

Dr Tim Forsyth-Jones

Dr Mevan Gooneratne

Dr Ryan Haines

Mr Shareef Madhi

Dr Rebecca Martin

Mr Hanzla Naeem

Mr Zak Rob

Dr Dan Sellers

Dr Tom Taylor

Dr Priya Thorat

Dr Sophie Walker

Dr Benjamin Jacobs

Dr Haren Jyothiraj

Dr Timothy McMillan

Dr Arun Menon

Dr Maria Muelmenstaedt

Dr Myura Nagendram

Dr Ching Pang

Dr Ashok Raj

Dr Daniel Wirth

Ms Kellie Allen

Dr Samuel Armanious

Mr Salvatore Bruni

Dr Keshava Reddy Burijinti Chenna

Dr Agilan Kaliappan

Ms Madelaine Ocampo

Ms Joanne Riches

Mr Louie Saclot

Ms Annaliza Sevillano

Mr Mark Vertue

Dr Adrian Percuin

Mrs Beena David

Dr Pushpaj Gajendragadkar

Dr George Gladstone

Dr Henry Hammerbeck

Mrs Marina Iaverdino

Dr Peter Knowlden

Ms Carina Lilley

Dr Ben Linton-Willoughby

Dr Daniel Murrell

Dr Shweta Patro

Dr Susanna Richie-Mclean

Dr Marc Turnbull

Dr Padmanabhan Vatsala

Dr Nick Black

Dr Ciara Coary

Dr Jonathan McCarter

Dr Killian McCourt

Dr Alexandra Murphy

Dr Raluca Ene

Dr Saba Iqbal

Dr Lizzie Irvine

Dr Christopher Perman

Dr Richard Pugh

Dr Iolo Roberts

Dr William Sutcliffe

Ms Stella Wright

Dr Linda Bairkdar

Dr Gillian Bennett

Dr Tom Bird

Dr David George

Dr Dhania Haron

Dr Chris Littler

Dr Roisin McCallum

Dr Benjamin O'Donovan

Dr Christopher Patrick

Dr Nowfal Rahman

Dr Tammy Towers

Dr Alicia Waite

Ms Annette Bolger

Dr Stephan Clements

Dr King Dhar

Ms Lucie Hobson

Ms Sion Lewis

Dr Michael Allan

Ms Cody Allen

Ms Nikkita Carden

Ms Amanda Cotterill

Dr Kerry Cullis

Dr Daniel Eden

Ms Nicola Farmer

Ms Andrea Galloway

Dr James Geoghegan

Dr Ahmed Gilani

Ms Kelly Hard

Dr Khalid Hasan

Dr Christopher Horner

Ms Virginia Iqbal

Ms Julie Lowe

Dr Ravindra Mallavalli

Ms Shanteela McCooty

Ms Sian McKillop-

Ms Diane Mellers

Dr Ronan Mukherjee

Dr Michelle Nicholas

Ms Chloe O'Hara

Dr Fiona Osborne

Dr Jane Pilsbury

Dr Emma Plunkett

Dr Gauhar Sharih

Dr John Westwood

Dr Annabelle Whapples

Ms Diane Whitehouse

Dr Lucinda Williams

Dr Bilal Yasin

Miss Janette Brown

Dr Jason Cupitt

Mrs Emma Stoddard

Dr Paul Athanasopoulos

Dr Madhu Balasubramaniam

Dr Mohammad Bhatti

Dr Laura Blood

Dr Hugo Buckley

Mrs Julie Chadwick

Mrs Shirley Cocks

Dr Deborah Fradkin

Mrs Emily Hetherington

Miss Rebecca Hill

Mr Rob Hull

Mrs Alison Loftus

Miss Emma McKenna

Dr Lucy Mcmanoman

Dr Lauren Milian

Mrs Raksha Mistry

Mrs Lisa Murthen

Mr Dave Parkinson

Miss Kat Rhead

Dr Peter Sandbach

Dr Ciara Walker

Dr Emma Wheatley

Dr Stephen Alderson

Dr Christopher Bull

Dr Rachel Butterworth

Dr Simon Cousins

Dr Sarah Goellner

Dr Mark Greasley

Dr Fiona Lyle

Dr Michael Munro

Mr Martin Northey

Dr Robert Spencer

Dr Stuart White

Dr Alex Harrison

Dr Paul-Simon Whitney

Dr Rohan Babla

Dr Ash Bharti

Dr Stephen Cole

Dr Jeremy Drake

Dr Anil Golhar

Dr Peter Indoe

Dr Rosada Jackson

Dr Mariam Latif

Dr Melvin Leong

Dr Matthew Maton-Howarth

Dr Victoria Millar

Dr Ben Millette

Dr Hannah Oliver

Dr Yasir Rashid

Dr Sana Rizvi

Dr Rosahn Saleh

Dr Harisg Venkatesh

Dr Stephen Crotty

Dr Amy Farrow

Dr Henry Lewith

Dr Vandita Ralhan

Dr Maria Rivero-Bosch

Dr Kate Wilson

Mrs Precious Basvi

Mrs Gillian Bell

Dr James Edwards

Dr David Lee

Dr Guanmei Luo

Miss Clare Mewies

Dr Ashok Nair

Dr Arif Qureshi

Dr Behzad Sohail

Dr Sachin Valap

Dr Deepti Bhuwanee

Dr James Chan

Dr John Dereix

Dr Allen George

Dr Tasmeen Ghafoor

Dr Emma Gold

Dr Daniel Haigh

Dr David Harding

Dr Stephen Hill

Dr Bhavia Janardhana

Dr Jithu Jayan

Dr James King

Dr Pnt Laloë

Dr Lauren Pearce

Dr Amit Pruthi

Dr Eleanor Roscoe

Dr Deepak Seharawat

Dr Mark Stubbington

Dr Vinayak Vanjari

Dr Anantharaman Venkataraman

Dr Serena Yen

Dr Charles Chan

Dr Garry Davenport

Dr Peter Featherstone

Dr Petrus Fourie

Dr Christopher Hall

Dr Preeti Mahidik

Dr Elisa Masoni

Dr Andrea Ortu

Dr Anita Patil

Dr Vishal Patil

Dr Janet Pickett

Dr Sandeep Sharma

Dr Lucy Allen

Dr Nathan Anderson

Dr Ethan Bateson

Dr Sanchita Bhatia

Dr Sara Churchill

Dr Leon Cohen

Dr Patrick Colhoun

Dr Charlotte Dunn

Dr Carys Durie

Dr Sarah Elgarf

Dr Jenny Ferry

Dr Naomi Goodwin

Dr Jeremy Guilford

Dr Claire Halligan

Dr Jade Harrison

Dr Dominic Hayes

Dr Ben Holst

Dr Rhys Hughes

Dr Rhys Hughes

Dr Thomas Hunt

Dr John Jackson

Dr George Kohler

Dr Michael Kriger

Dr Svetlana Kulikouskaya

Dr Amit Kurani

Dr Charlotte Maden

Dr Alex Moore

Dr Adam Mounce

Dr Alice O'Donnell

Dr Robert Sparrow

Dr Jayne Sutherland

Dr Mahmoud Alkholany

Dr Zoe Apple

Miss Anam Asif

Dr Rebecca Coates

Dr Liliana Czukowska

Dr Lauren Elliott

Dr Li Fang

Dr Hannah Greenlee

Dr James Hanison

Mrs Jashmin Maria

Dr Tarek Mostafa

Dr Sujesh Bansal

Dr Neeraj Bhardwaj

Dr Karl Braid

Dr Nick Greenwood

Dr Amarjeet Patil

Mrs Christine Adamson

Mrs Carina Bautista

Miss Rhian Bull

Mr Jaime Carungcong

Miss Elna Cifre

Dr Olivia Clancy

Dr Lauren Friedman

Dr Gabriella Frunza

Miss Hindusha Keerthikumar

Dr Helen Laycock

Dr Sarah Leir

Mrs Carmela Martella

Miss Minna Meritahti

Mrs Kribashnie Nundlall

Dr Kirat Panesar

Dr Eleanor Pett

Dr Catriona Routley

Dr Amee Samani

Dr Vatsharlan Santhirapala

Dr Alice Sisson

Mrs Mini Thankachen

Dr Charlotte Topham

Miss Sathya Visvendra

Dr Anna Warrington

Dr Andrea Weigert

Dr Helen Wibberley

Dr Philip Barclay

Mrs Amy Barker

Mr Suman Biswas

Dr Fenner Christoper

Dr Sadia Habib

Mrs Ursula Kirwan

Mr Victor Maduekwe

Dr Alexandra Matson

Mr Metod Oblak

Dr Timothy Peters

Mrs Amrinda Sayan

Mrs Marie-Louise Svensson

Mrs Bernadette Tilley

Dr Mia Andrews

Dr Sarah Beavis

Dr Daiva Bernotaitis

Dr Shalini Chinna

Dr Helen Church

Ms Sian Edwards

Ms Nicky Ford

Ms Sarah Hazeldine

Dr James Hudson

Ms Claire Macey

Dr Matthew Morgans

Dr Nick Spittle

Ms Julie Toms

Ms Amanda Whileman

Mrs Jan Woodward

Ms Stephanie Wright

Mrs Ashley Allan

Dr Bryony Burrill

Dr Lynn Fairless

Dr Nicholas Francis

Dr Colin McAdam

Miss Julie Sheriff

Dr Marc Slorach

Dr Shayan Arshed

Dr Clare Bird

Ms Debbie Campbell

Mrs Elaine Chinery

Dr Liam McLoughlin

Dr Enoch Onya

Dr Ryan Perry

Dr Jose Miguel Sabugueiro

Dr Lydia Shatanda

Dr Muzaffar Sheik

Dr Jo simpson

Dr Gabriela Wong

Dr Chen Yun-Han

Dr Duncan Adshead

Dr David Castillo

Mrs Maria Faulkner

Dr Kim Gibson

Dr Tara Keogh

Dr Sam Michlig

Dr Elizabeth Perritt

Dr Simon Ridler

Dr Kate Tizzard

Dr Anne Troy

Dr Lawrence Wilson

Ms Sarah Clark

Sister Amanda Cowton

Dr Karin Duckett

Dr Tom Johnson

Dr M. Amir Rafi

Mr David Rollins

Ms Asia Sarwar

Ms Julie Temple

Ms Jane Varin

Mr Martin Warin

Mrs Vikki Atkinson

Dr Tariq Azad

Dr Vladimir Bashliyski

Mrs Ellen Brown

Mrs Jill Deane

Mrs Jean Dent

Mrs Louise Duncan

Mr Nicholas Hooper

Mrs Andrea Kay

Dr Donna Kelly

Mrs Melanie Kent

Dr Jo Knight

Dr Christina McCarroll

Dr Helen Melsom

Miss Annie Newby

Mrs Kathryn Potts

Dr James Roe

Dr Lewis Schofield

Dr Deborah Skelton

Dr Sameer Somanath

Dr Peter Taysum

Dr William Udall

Dr Lucy Venyo

Mrs Shelly Wood

Dr Srinivasan Dhileepan

Dr Ravishankar Jakkala Saibaba

Dr Emma Stewart

Dr Eleanor Warwick

Dr Rhys Griffiths

Dr Suzi Hale

Dr Ceri Lynch

Dr Ceri Lynch

Dr Abbas Majeed

Dr Ifan Patchell

Dr Jennifer Quinton

Dr Babak Sedghi

Dr Stephanie Wallis

Dr Maryna Garmash

Dr Fatma Lahloub

Dr Omar Pemberton

Dr Zakaulla Belagodu

Dr Shirin Dastur

Ms Bridget Fuller

Mrs Imogen Hayes

Dr Ashwini Keshkamat

Dr Georgia Monantera

Ms. Wakefield Phillipa

Dr Roxana Sandhar

Dr Mansoor Sange

Dr Shamini Sivakumaran

Dr Megan Thomas

Dr Manish Torne

Mrs Sherma Turner

Mrs Catherine Addleton

Mrs Lisha Aju

Mrs Marie Appleby

Mrs Elizabeth Barnes

Mrs Trish Boateng

Dr Laura Carrick

Mrs Elaine Coulborn

Miss Rebecca Darbyshire

Mrs Charlotte Downes

Miss Jodie Fitzgerald

Mrs Linda Hall

Mrs Laura Harvey

Dr AMR HASSAN

Mrs Kathleen Holding

Miss Lianne Hufton

Mrs Louise Humphries

Mr Ryan Humphries

Mr Laurence Inman

Mrs Nicole Issit

Mrs Sarah Longhurst

Mrs Melody MacGregor

Mrs Trusha Mistry

Mrs Mona Mohamed

Miss Charlene Otieno

Dr David Read

Dr David Rogerson

Miss Michelle Scott

Miss Abbie Singleton

Mrs Jill Smith

Miss Aariana Sohal

Mr Samson Tou

Mrs Vanessa Unsworth

Mr Richard Wan

Mr Christopher Worth

Dr Rose Buckley

Miss Rachel Codling

Dr Richard Dobson

Mr Cain Hunter

Dr Jenny Jackson

Dr Caroline Lowrie

Dr Catherine McMillan

Dr Raj McNab

Dr Max Richardson

Dr James Sylvester

Dr Anna Watkin

Mrs Rachel Bown

Dr Jonathan Chambers

Mr Colin Christie

Ms Stephanie Dukes

Mrs Sarah Horton

Mr Andrew Rees

Mrs Patricia Williams

Mrs Sarah Williams

Charge Nurse Catherine Jardine

Dr Graham White

Dr Dewi Williams

Dr David Wright

Miss Chanice Alcock

Dr Wei Lin Allen

Mrs Pearl Baker

Mrs Hannah Beadle

Dr Jon Bramall

Dr Radu Chirvasuta

Miss Amina Chohan

Mrs Angela Christofides

Mrs Angela Cook

Mrs Janet Cotta

Miss Carina Cruz

Mrs Jemma Gilmore

Mrs Vicky Hills

Dr Sunil Jamadarkhana

Dr Kathryn King

Dr Lucy McClelland

Dr Sei Nishimura

Mrs Rumyana Nyathi

Dr Prasan Panagoda

Mrs Louise Peacock

Mrs Emma Shinn

Miss Sophie Spencer

Mrs Mayumi Vianzon

Dr Ritoo Kapoor

Dr Vanja Srbljak

Ms Tracey Cosier

Dr Walid Hammad

Ms Tracy Hazelton

Mrs Angela Moon

Ms Janine Musselwhite

Dr Nagendra Natarajan

Mr Joshua O' Donnel

Mr Joshua O'Donnell

Dr Pushkar Patankar

Dr Richard Stead

Dr Ismail Tariq

Ms Sharon Turney

Dr Freda Amoakwa-Adu

Dr Sinan Bahlool

Ms Sarah Driscoll

Dr Claudia Dulea

Dr Andrew Feneley

Dr Marcus Fletcher

Dr Kim Hoyland

Dr Bennur Katyayani

Dr Catherine Lloyd

Dr Joanna Moore

Dr Ganesh Nair

Mr Joshua O'Donnell

Dr Katy Redington

Dr Aalia Sange

Dr Mark Snazelle

Dr Janine Thomas

Dr Zackriah Badsha

Ms Sam Bews

Dr Kumud Bhandari

Dr Greg Cox

Ms Jill Fitchett

Dr David Freeman

Dr Asif Gani

Ms Bev Hammond

Dr Ramez Ibrahim

Dr Brian Johnston

Dr Istvan Koczka

Dr Jason Lie

Mr Matt Milner

Ms Nicky Moss

Dr Jeet Patel

Dr Gillian Rennie

Ms Karen Riley

Ms Catherine Cartmell

Dr Srikanth Chukkambotla

Hawa Desai

Dr Mark Doran

Mr Stephen Duberley

Ms Diane Forrest

Ms Catherine Gedling

Ms Jessica Giles

Ms Wendy Goddard

Ms Linda Gregson

Ms Yvonne Grimes

Ms Janice Hartley

Ms Gemma Hudson

Nasreen Iqbal

Ms Karen Jewers

Dr Abhishek Kakkar

Mr Matt Lovell

Dr Mohamad Mahmoud

Dr Aji Thomas Mathew

Ms Cathie Melvin

Dr David Perry

Dr Mike Pollard

Dr Stephanie Reed

Dr Sherrie Samuels

Dr Ioana Simionescu

Dr Lorna Sissons

Dr Stephen Traynor

Dr Daniel Tucker

Dr Glenn Vetuz

Ms Gillian Whalley

Dr Tim Arnold

Dr Jamie Brookes

Mrs Anne Cowley

Dr Ruth Delascasas

Mrs Emma Edmunds

Dr Dominic Espitalier-Noel

Dr Rajeev Jeevananthan

Mrs Jenni Law

Dr Philippa Marshall

Mrs Kelly Mintrim

Dr Jack Reid

Mrs Jackie Terry

Dr Manish Verma

Dr Jean-Paul Zahra

Dr Lindsay Dawson

Dr Clare Donovan

Dr Jo Han Gan

Dr Andrew Selman

Dr John Bailes

Dr Kaya Jeyarajah

Dr Geoff Thorning

Dr Yin Yong Choo

Dr Mihir Desai

Dr Sindy Lee

Dr Justin Woods

Dr Peter Csabi

Mrs Saheli Das

Mrs Clare Denford

Dr Rahul Dimber

Dr Khaled Ellisy

Dr Ben Griffiths

Dr Alex Daniels

Dr Vanessa Linnett

Dr Sadie Perkin

Sister Jenny Ritzema

Sister Bryony Storey

Dr Christine Wood

Dr Charis Banks

Dr Will Gatfield

Dr Tom Hickish

Dr Emily Hignell

Dr Jo Mullender

Dr Robert Orme

Dr Kath Rosedale

Dr Milena Vannahme

Dr Oliver Barker

Mrs Susan Beames

Dr Aislinn Brown

Dr Lucy Corbett

Dr James Dalton

Dr Fiona Davis

Mr Matthew Edmunds

Dr Edmund Gerrans

Mrs Paula Hiltout

Dr Helen Johnston

Dr Sock Koh

Mr Sam Miller

Mrs Susan O'Connell

Dr Katie Samuel

Dr Gary Baigel

Ms Sarah Grayland

Dr Victoria Hawley

Ms Laura Mc affrey

Ms Suzannah Peggler

Mr Joe Stevens

Ms Yemi Adelaja

Dr Elsie Bickmore

Dr Heena Bidd

Dr Jenny May-Ling Cheung

Ms Ndi Ekwere

Dr Kariem El-Boghdadly

Ms Ange Lise John-Baptiste

Ms Natasha Muzengi

Dr Cheng Ong

Dr Maryam Zaky

Dr Sanjoy Bhattacharyya

Dr Suzanne Body

Dr Priaykam Chowdhury

Dr Joshua Cuddihy

Dr Paul Kelly

Dr Daniel Leslie

Dr Wint Mon

Dr Nisha Pattni

Dr Thomas Potter

Dr Elena Stanton

Dr Liana Zucco

Miss Denise Griffin

Dr Heidi Lightfoot

Mr McDonald Mupudzi

Miss Katherine Nahajski

Dr Segun Oladele

Dr Richard Partridge

Dr Ian Sheldrake

Dr Patrick Tapley

Dr Nimu Varsani

Dr Eleanor Walshe

Dr Helen Bromhead

Dr Peter Evans

Dr J. Joseph Kinsella

Dr Alexander Knight

Dr Rosemary Anna Lewis

Ms Jane Martin

Dr Julia Ottaway

Dr Stephen Petley

Dr Alexander Michael Stewart

Dr Aaron Stokes

Ms Dawn Trodd

Ms Caroline Wrey Brown

Dr Natalie Baldry

Ms Caroline Bennett

Dr Sophie Earl

Ms Joyce Guy

Dr Martin Huntley

Mrs Maggie Peat

Mrs Lorraine Stephenson

Dr Hao Ern Tan

Mrs Louise Wills

Ms Michelle Yare

Dr Supriya Antrolikar

Dr Fayaz Baba

Ms Hollie Bancroft

Ms Mary Bellamy

Ms Mirriam Sangombe

Ms Katie Atterbury

Ms Safia Begum

Dr Keith Couper

Ms Lucy Evans

Ms Rachel Flight

Dr Catriona Frankling

Dr Srikant Ganesh

Ms Joanne Gresty

Ms Salma Kadiri

Dr Aalisha Mariam Karimi

Ms Amy Kerr

Ms Faye Moore

Dr Jennifer Morrish

Dr Vanisha Patel

Ms Eleanor Reeves

Dr Richard Robley

Ms Julia Sampson

Mr Vusumuzi Shabangu

Ms Lucy Sheppard

Mr Peter Sutton

Ms Joanne Taylor

Dr James Turner

Dr Rochelle Velho

Ms Joanne Webb

Ms Linda Webber

Dr Ewa Werpachowska

Ms Lisa Wilkinson-Guy

Dr Joyce Yeung

Ms Ruth Joslyn

Ms Teresa Melody

Ms Mary O'Sullivan

Ms Sam Stafford

Dr Ayman Abdu

Ms Helen Bowyer

Dr Ulf Buhmann

Miss Victoria Christenssen

Dr James Collins

Miss Alice Groves

Dr Jyothi Hosahalli

Dr Dancho Ignatov

Dr George Koshy

Dr Robyn Lee

Mrs Susan Martin

Dr Piers Murphy

Mrs Pamela Oracki

Mrs Tara Pauley

Dr Panagiotis Sgardelis

Dr Tom Stocks

Dr Sivaprakash Vaitheeswaran

Dr Priya Verma

Dr Madlena Ivanova Vrazhalska

Dr Hari Arunachalam

Dr Victoria Ashton

Dr Jens Full

Dr Samira Green

Dr Chiraag Talati

Mrs Caroline Abernethy

Dr Abigail Clarke

Dr Pallavbhai Desai

Dr Andrew Gratrix

Mrs Victoria Martinson

Dr Ravi Parekh

Dr Mirain Phillips

Dr Anju Raina

Dr Sarah Raut

Dr Chris Smales

Mr Neil Smith

Dr Ruth Ugochukwu

Dr Matt Willis

Dr Sunita Agarwal

Dr Randeep Dhaliwal

Dr Hywel Evans

Dr Ahmed Foly

Dr Ahmed Hassanin

Dr Ahmad Huda

Dr Ilona Schmidt

Dr Norbert Skarbit

Dr Brian Campbell

Dr Maggie Collingborn

Dr Zhana Ignatova

Dr Ramesh Khoju

Dr Maria Lackmann

Dr Christopher Nwaefulu

Dr Obla Suganthi

Dr Jacek Zeber

Dr Harry Barclay

Dr Richard Dagnan

Dr David Steven Davies

Dr Ishan Dharmarathna

Dr Yuvraj Doriaswami

Dr John Francis

Dr Stuart Gill

Dr Tim Green

Dr Peter Havalda

Dr Beryl Jones

Dr Kofi Mensah

Dr Gopinath Selvraj

Dr Glenn Arnold

Dr James Carvell

Dr Anthony Carver

Dr Fiqry Fadhlillah

Dr Vineetha Jayakumar

Dr Lara Jeanes

Dr Jasmine Jose

Dr Emma Karsten

Ms Stephanie Lewis

Dr Dafydd Lloyd

Mrs Francisca Mautadin

Dr Claire McCahill

Ms Susan McInerney

Dr Elaine Morsman

Dr Vidhya Nagaratnam

Dr David O'Callaghan

Mrs Bhavesh Pratap

Dr Stephen Smith

Ms Maie Templeton

Dr Rebecca Wilson

Dr Claire Boynton

Dr Stephen Brett

Dr Matt Clayton

Ms Keyury Desai

Dr Mhairi Jhugursing

Ms Laura Morland

Dr Smitangshu Mukherjee

Ms Emily Pickford

Ms Alka Shah

Dr Sophie Uren

Dr Christiana Georgiou

Dr Susan Kirby

Dr Flora Kormendy

Dr Chandana Rao

Dr Kavita Upadhyaya

Dr Daniel Bendel

Dr Sunny Nayee

Dr Claire Smyth

Dr Icel Souleimanova

Dr Shree Voralia

Dr Justin Ang

Mrs Debroah Beeby

Mrs Stephanie Bell

Dr Helen Boys

Miss Sue Brixey

Mrs Cathleen Chabo

Dr Lynsey Cubitt

Ms Maggie Dawson

Mrs Jenny Finch

Dr Vlad Kushakovsky

Dr Robert Lewis

Mrs Bally Purewal

Dr Kaung Pyae

Ms Judi Ramsey

Dr Ben Scoones

Dr Gabor Debreceni

Dr Obaid Tarin

Ms Nicola Zondo

Dr Pieter Bothma

Dr Jack Carmichael

Miss Teresa Ferreira

Miss Kelly Goffin

Dr Sarada Gurung

Dr Ashish Kundu

Dr Sarah MacLennan

Dr Theresa Murray

Dr Karan Verma

Dr Graeme Wilson

Dr Aleinmar Winthein

Dr Muneeba Ahmed

Dr Duncan Baines

Mrs Charmaine Beirnes

Miss Chrissy Braybrook

Miss Olga Fernandez

Mrs Jayshree Gracey

Dr Snehasish Guha

Dr Arihant Jain

Dr Satyanarayan Jakkampudi

Dr Raghavendran Krishnaiyan

Dr Thomas Moody

Mrs Jo Novaga

Dr Frances Tait

Miss Jemma Tate

Dr Jennifer Claire Taylor

Miss Molly Waldron

Mrs Sonia White

Dr Mohamed Ahmed

Dr Lizzie Ashton

Dr Andrew Robert Bailey

Dr Pele Banugo

Ms Sian Birch

Dr Alice Brown

Dr Mark Chen

Dr Lucy Connolly

Dr Alexandra Edwards

Dr Timothy Faccini

Dr Theodore Floyd

Dr Thomas Georgiou

Dr James Gill

Dr Anish Gupta

Dr Philip Hopkins

Dr Rhiann Marie O'Shaughnessy

Dr Laura O'Sullivan

Dr Seema Pai

Dr Benjamin Parsons

Dr Laura Peltola

Ms Beth Penhaligan

Dr Merate Place

Dr Jonathan Ramsden

Dr Kathy Shammas

Dr Ali Watts

Dr Valantine Woodham

Dr Claire Botfield

Dr Henry Boyle

Dr Viral Dalal

Dr Shaima Elnour

Dr Russell Hedley

Dr Roshmi Kumar

Dr Alice Michell

Dr Gamunu Ratnayake

Dr Thomas Williams

Ms Jennifer Crooks

Dr Ania Dean

Dr Chris Honstvet

Dr Katherine Hunter

Dr Sarang Puranik

Dr Sarah Steynberg

Mr Andrew Swain

Dr Alex Bonner

Ms Donna Doyle

Dr Robert Fallon

Dr Shondipon Laha

Mr Mark Verlander

Miss Alexandra Williams

Dr Richard Yardley

Dr Sheila Black

Dr Robert Collin

Mr Gareth Harrop

Dr Peter Hart

Mrs Clare Howcroft

Dr Carl Ilyas

Dr James Morgan

Dr John Stones

Dr Zaid Ahmed

Dr Ossian Aukland Child

Mr Jamie Calderwood

Dr Andy Chapman

Ms Kinga Dwornik

Dr Kate Gallagher

Dr Michelle Gardener

Dr Umakanth Kempanna

Dr Gnanshree Krishnamurthy

Dr Paramesh Kumara

Dr LI Lian Loh

Dr Jonathan Pipe

Dr Anand Sathiapillai

Dr Simon Whiteley

Mrs Elizabeth Wilby

Dr Tatyana Bolonenkova

Dr Lisa Dunlop

Dr Jenna Kelly

Dr Mitul Patel

Dr Charles Prior

Dr Katie Rowland

Dr Buzz Shephard

Dr Jennifer Van Ross

Dr Manju Agarwal

Dr Karthick Duraisamy

Dr Eveliina Nurmi

Dr Jonathan Perry

Mrs Rosie Reece-Anthony

Dr Bernd Oliver Rose

Dr Helen McNamara

Dr Natasha Permall

Dr Gillian Robertson

Dr Sarah Siew

Miss Amy Smith

Ms Sue Spearritt

Dr Dipali Verma

Dr Rhys Williams

Dr Samantha Clayton

Dr James Goddin

Dr Stuart Joy

Dr Vikram Malhotra

Dr Samuel Mindel

Dr Zoe Neilson

Dr Shanelle Tharuka Wijesuria

Dr Ryan Wilkins

Dr Suhail Zaidi

Dr Andrew Bradley

Mrs Samantha Coetzee

Dr Alexandra Crook

Mrs Eunice Emeakaroha

Dr Marek Frenkiel

Mr Tom Hatton

Dr Kootharajan Kamraj

Miss Natalie Long

Dr Badrinath Manikundalam

Dr Kirtida Mukherjee

Dr Muditha Peiris

Ms Catherine Plowright

Dr Matthew Roche

Mr Banher Sandhu

Dr Nichola White

Mrs Annette Woods

Dr Adam Yarnold

Dr Marion Ashe

Dr Tom Disney

Ms Caroline Dixon

Dr Emily Dodds

Dr Sarah El-Sheika

Dr Yasir Hameed

Dr Rob Jones

Dr Karan Kanal

Dr Nicolas Price

Dr Sanjeewa Ranaweera

Dr Simon Ripoll

Dr Nicki Russell

Ms Sally Anne Smith

Dr Swee Ang Tung

Ms Victoria Apps

Ms Mandy Austin

Dr Laura Catchpole

Mrs Christine Catley

Miss Jenny Child

Mrs Karen Cranmer

Ms Lizzie Dawson

Dr Bruce Emerson

Miss Beth Farr

Ms Helen Gerrish

Ms Sian Gibson

Ms Jacqueline Gunn

Mrs Kim Holland

Ms Lorri James

Dr Dushyanthi Jayasekera

Ms Yvonne Lester

Mrs Amanda Lyle

Ms Fiona McNeela

Dr Aneta Oborska

Dr James Pennington

Ms Lauren Perkins

Mrs Hollie Robinson-Perrie

Mrs Lauren Shillito

Mr Bryan Singizi

Ms Susan Smolen

Mrs Joanne Topliffe

Mr Ben Vowles

Ms Natalie Whybro

Amon Wijunamai

Mrs Sarah Williams

Ms Lucy Willsher

Ms Joanne Wootton

Dr Philip Atkinson

Mrs Sarah Buckley

Mrs Thelma Darian

Miss Sachini Dhamaratne

Dr Richard Gould

Dr Laura Graham

Miss Charlotte Hirst

Mr Andrew Peeling

Dr Saxon Prentice

Dr Naresh Rajasekar

Dr Alastair Rose

Dr Craig Smith

Dr Sandeep Varma

Dr Kate Wilkinson

Ms Chrissie Chevis

Dr John Ekpa

Nurse Sara Greig

Nurse Rebecca Hinch

Ms Carole Holder

Dr Nikolaos Makris

Nurse Louise Moran

Nurse Cheryl Padilla Harris

Nurse Diane Scarletta

Ms Jeanette Smith

Dr Richard Stewart

Nurse Sara-Beth Sutherland

Ms Joanne Turner

Dr Peter Valentine

Dr Amelia van Manen

Nurse Sonia Walia

Nurse Charlotte White

Dr Georgina Wilson

Nurse Lynn Wren

Nurse Francesca Wright

SN Debbie Callaghan

SN Danielle Gilmour

Dr Peter O'Brien

Dr Ruth Clarke

Dr Stephen Hickey

Dr Stephanie Hii

SN Megan McAtear

SN Sharon Meehan

Dr Gemma Milne

Dr James Small

Dr Graeme Foggo

Dr Susan Livingstone

Ms Megan McAtear

Ms Sharon Meehan

Dr Joellene Mitchell

Dr Gillian Fleming

PA-A Marc Hastie

Dr Catherine Hunter

Dr Jennifer Lockhart

Dr Kirsten Reid

Dr Hilary Robb

Dr Richard Appleton

Mr Michael Briskoe

Dr Fiona Christie

Dr Shub Gupta

Dr Colin Hall

Dr Jane Hamilton

Dr Johann Harten

Dr Laura Hunter

Dr Gregor Imrie

Dr Euan Kerr

Ms Aillison MacLean

Dr Katherine McDowall

Dr Finbar O'Sullivan

Dr Sarah Ramsay

Dr Calum Taylor

Mr James Woodier

Dr Richard Cowan

Dr Barbara Crooks

Dr Alice Drysdale

Dr John Gardner

Dr Michael Kinsella

Dr Gemma Scotland

Dr Alvin Soosay

Dr Charlotte Soulsby

Dr Sara Stevenson

Dr Caroline Thompson

Dr Robin Williams

Ms Maya Kommer

Professor James McCaul

Dr Graeme Finnie

Dr Suleman Mulla

Dr Manfred Staber

Dr Graeme Brown

Dr Stewart Brown

Dr Guy Coady

Dr Stewart D'Sylva

Dr Paul Edgar

Dr Umairali Ikram

Dr Brian Lafferty

Dr Tom Lovejoy

Dr Maria Loy

Dr David MacPherson

Dr Cara Marshall

Dr Ruth Mawhinney

Dr Claire McAteer

Dr Michael McCusker

Dr Christopher McGovern

Dr Stewart Mckie

Dr Melanie Morrison

Dr Cristina Niciu

Dr Su Ying Ong

Dr Sophie Parcell

Dr Tom Pettigrew

Dr Urmila Ratnasabapathy

Dr Rebecca Robson

Dr Nithin Roy

Dr Jasmine Samuel

Dr Natasha Sharma

Dr Kate Slade

Lois Steuart

Dr Laura Sweeney

Dr Kerwei Tan

Dr Francesca Th'ng

Dr Daphne Varveris

Dr James Wardlow

Dr Martin Watson

Dr Simon Young

Dr Michael Brett

Dr Christopher Lochrin

Dr Elizabeth Neale

Dr David Robinson

Dr Scott Berwick

Dr Andrew Boyle

Dr Neil Brown

Dr Anna Celnik

Dr Alistair Coleman

Dr Mohamed Elwkhiee

Dr Mario Fernandes

Dr Katie Hanlon

Dr Jacqueline Howes

Dr Sarah MacLean

Dr Rhian Morgan

Dr Sunny Bhat

Dr Rachel Campbell

Dr Anna Cormack

Dr Jonathan Edgar

Mrs Laura Ferguson

Mrs Donna Ferraioli

Dr Moyra Hynd

Dr Adam Janeczko

Dr Sally Jeffrey

Dr Tony Kinsey

Dr Emma Murphy

Miss Kris Parker

Dr Tom Reevell

Miss Fiona Robertson

Dr Will Shankey-Smith

Dr Stuart Watson

Dr Yvonne Bramma

Dr Roddy Chapman

Dr Karen McIntosh

Dr John McKenna

Dr Ryhs Millington

Dr Ali Atrah

Dr Euan campbell

Dr Stephan Dalchow

Dr Ammy Dodd

Dr Yuvaraj Kummur

Ms Maria Mclaughlin

Dr Khaled Razouk

Dr Colum Slorach

Dr Tamas Szelei

Dr Geoff Warnock

Dr R Peter Alston

Dr Karen Birnie

Dr Julia Critchley

Dr Callum Forbes

Dr Helen French

Dr Carolyn Smith

Dr Robert Stuart

Dr Murray Geddes

Dr Maximiliane Kellner

Dr John McLenachan

Dr Hannah Phelan

Dr Fraser Waterson

Dr Elizabeth Wood

Dr Eireann Allen

Dr Kirsteen Brown

Dr Margaret Cullen

Dr Alex Eros

Dr David Hall

Dr Jonathan Hetherington

Dr Keith Kelly

Dr Hiba Khaled

Dr Damien Mantle

Dr Stuart McLellan

Dr Susan Midgley

Dr Susan Nimmo

Dr Bart Ordys

Dr Philip Roddam

Dr Louise Shaw

Dr Elizabeth Steel

Dr John sturrock

Dr Alasdair Waite

Dr Hannah Watson

Research Nurse Elizabeth Boyd

Dr Surabhi Jain

Research Nurse Elaine Matthews

Dr Geejo Rappai

Prof Stefan Schraag

Dr Catriona Barr

Dr Michael Stewart

Dr Kate Arrow

Dr Pauline Austin

Dr Susie Chapman

Dr Sharon Christie

Dr William Malein

Dr Amy Sadler

Dr Jonathan Little

Dr Tim Smith

Dr Rhona Younger

Dr Katy Allan

Dr Vignesh Ashok

Ms Ayda Borjian Boroojeny

Mr John-Paul Cutts

Dr Fiona Faulds

Mr Mark Fernie

Dr Amit Gadre

Dr Tom Gately

Dr Richard Haddon

Dr Lalani Induruwage

Dr Siobhan King

Dr Sian Liddle

Dr Paul Mallett

Dr Saurabh Mehotra

Dr Inthekab Mohammed Ali

Dr Rachel Morris

Dr David Nunn

Dr Rocio Ochoa Ferraro

Dr Pulak Padhi

Dr Ewa Prusack

Dr Edmund Quak

Dr Caroline Reavley

Dr Georgina Singleton

Dr Kin So

Dr Daniel Stolady

Dr Drew Welch

Dr Michael Whitear

Dr Kathy Wilkinson

Dr James Wu

Dr Alex Yusaf

Dr Ewen Cameron

Dr Lynn Fenner

Dr Jake Hartford-Beynon

Dr Sarah Martindale

Dr Iain Mooney

Dr Charlotte Mundy

Dr Rhys Rhidian

Dr Sophie Scutt

Dr Anna Todd

Ms Amy Ashton

Mrs Theresa Cooper

Ms Katherine Davidson

Mrs Anna McSkeane

Dr Mahamed Mostafa

Dr Gururaj Mudimadagu

Dr Timothy David Smith

Ms Antoinette Wilson

Dr Suresh Eapen

Dr Fiona Graham

Ms Crowther Hannah

Ms Claire Hindmoor

Ms Polly Rice

Dr Hemangini Barot

Dr Shady Elhallous

Dr Ada Ezihe-Ejiofor

Dr Rizana Ghafoor

Dr Vitul Manhas

Dr Tanmay Patil

Dr Nitin Sadavarte

Dr Bhamini Tharmalingam

Ms Hayley Bridger

Ms Jean Bage

Dr Sarah Higgin

Dr Asokan Krishnaier

Dr Esther Neilly

Dr David Pritchard

Dr Sean Rayappu

Dr Chye Siaw

Dr Ilma Songaile

Dr Peter Standen

Dr Narayanan Suresh

Dr William Weston

Ms Lynne Williams

Dr Jiang Yuchen

Dr Hoda Abou Ghoneim

Mrs Sara Balliston

Mrs Lucy Dudgeon

Dr Muna Elsheikh Idris

Dr Prashant Kakodkar

Dr Juneenath Karattuparambil

Dr Lohita Nanda

Dr Bhavesh Raithatha

Dr Mahmood Saad

Mrs Jenny Spimpolo

Mrs Linda Titinchi

Mrs Julie Wilson

Mrs Ulrika Winstone

Dr Garry Henry

Dr Nigel Hollister

Mrs Jane Hunt

Mrs Amanda Isaac

Dr David Morris

Mr Antonio Paredes- Guerra

Dr Katherine Pass

Dr Martin Paul

Dr Kiran Rait

Dr Guy Rousseau

Dr Emma Shacklock

Mrs Amanda Skinner

Mrs Lucia Stancombe

Dr Laura Thomson

Dr Johnny Holland

Dr Christopher Nutt

Mrs Orla O'Neill

Dr Arun Sengottaiyan

Dr Geoff Wright

Dr Temitope Aiyedun

Dr Mark Clayton

Ms Lisa-Jayne Cottam

Dr Elizabeth Evans

Mr Jonathan Hatton

Dr Jason Mann

Dr Atideb Mitra

Mrs Maria Newton

Dr Jaspreet Rayet

Dr Rebecca Saunders

Dr Jonathan Adams

Dr Hani Ali

Dr Sahar Biuk

Miss Bryany Bond

Mrs Michelle Cheeseman

Mrs Kathy Dent

Mrs Sandra Evans

Dr Sanjeev Garg

Miss Hannah Goodhand

Mrs Karen Green

Mrs Joanne Hill

Mrs Dorothy Hutchinson

Dr Shibu Jacob

Dr Renjith Joseph

Dr Laura MacNally

Mrs Virginia McTaggart

Mrs Sandra Pearson

Dr Sandeep Saxena

Dr Hemamangala Venkatesh

Mrs Nikita Whotton

Dr Ravi Alagar

Dr Brian Yates

Miss Claire Matata

Dr Roopa McCrossan

Dr Andrew Robertson

Dr Priya Shekar

Ms Wendy Stoker

Dr Adrian Taylor

Dr Elizabeth Turnbull

Dr Clare Watkinson

Dr Stephen Worthy

Dr Bryan Yates

Dr Subha Arunachalam

Ms Zorba Begum

Dr Gemma Bown

Dr Luff Delme

Dr Stephen Hackett

Dr Richard Jones

Dr Arnab Mandal

Ms Shirley Pyke

Dr Sonia Rasoli

Dr Suneal Sharma

Ms Gemma Squires

Dr Peter Tsim

Dr Jonathan Veitch

Dr Helen Fenner

Dr Sonia Bhangu

Dr Avninder Chana

Dr Puja Chhaniyara

Dr Natalie Constable

Dr Oliver Griffith

Dr David Hewson

Dr Kristofor Inkpin

Dr Richard Kirkdale

Dr Ian Lyons

Dr Prerna Mehrotra

Prof Iain Moppett

Dr Martin Murphy

Ms Alison Pearce-Smith

Dr Martin Priestley

Dr Sam Clark

Dr Soumi Ghosh

Dr Nicholas Hingley

Dr Richard Siviter

Dr John Allen

Dr Linden Baxter

Ms Sally Beer

Ms Mariana Bernardo

Mr Giles Bond-Smith

Ms Holly Coles

Dr Jo Cudlip

Dr Talitha Devries

Dr Phil Dugglby

Ms Alexis Espinosa

Dr Karina Fitzgibbon

Dr Joao Galente

Ms Domonique Georgiou

Dr Olivia Kay

Dr Ryan Kingan

Mr Chris Levett

Mr Jose Martinez

Dr Alex Mattin

Dr Anna Moore

Dr Samuel Morrish

Dr Amy Nash

Dr Tejuswi Patel

Dr Cieron Roe

Dr Victoria Thwaites

Dr Anuj Wali

Dr Dafydd Watterson

Dr Anna Wilson

Dr Charlotte Yates

Dr Anita Boltres

Dr Marc Gimenez

Ms Rebecca McClean

Mr Sam Papadopoullos

Dr Elena Teh

Dr Stephen Webb

Dr Andrew Drummond

Mr Asad Javed

Mrs Linda Kent

Mrs Lorraine Lock

Miss Alison Meadows

Mrs Paula Mulligan

Mrs Maria O'Callaghan

Mr Jonathan Ogor

Dr Andrew Brammar

Miss Dionne Dervin

Dr Naomi Fleming

Miss Sanniah Hussain

Dr Katy Irwin

Mrs Denise McSorland

Dr Wendy Nichols

Dr Moiz Alibhai

Dr Daisy Alston

Dr Jolyon Cohen

Dr Laura Coleman

Dr Simon Davies

Dr Rebecca Dooley

Dr Douglas Findlay

Dr Yazzim Hammoud

Dr Joanne Humphreys

Dr Joanna Poole

Dr Geoffrey Ryder

Mr Akmal Shakoor

Dr Wei Teo

Dr Donata Banni

Dr David Brooks

Dr Matthew Davies

Dr Aaron D'Sa

Dr Steven Forde

Dr Lauren Hunt

Dr Angus McKnight

Dr Henry Nash

Dr Bassey Nkanang

Mr Alan Pope

Dr Romit Samanta

Dr Mike Weisz

Dr Stephen Adshead

Dr Helen Anderson

Dr Adam Carpenter

Dr Stuart Clelland

Dr Priya Datar

Ms Esme Elloway

Dr Matt Holl

Dr Aidan Hulbert

Dr James Humphreys

Mr Ben Hyams

Dr Sam McAleer

Dr Gary Minto

Dr Mary Newmarch

Ms Holly Notman

Ms Abigail Patrick

Dr Anna Perham

Ms Catherine Pitman

Ms Fiona Reed

Dr Paul Sampson

Ms Hannah Smith

Dr Sam Spinney

Ms Nina Toms

Dr Tim Warrener

Mrs Helena Barcraft-Barnes

Mrs Julie Camsooksai

Ms Carolyn Colvin

Dr Jack Davies

Dr Michael Girgis

Dr Alex Hamilton

Dr Fiona Linton

Dr Greg Nussbaum

Ms Sarah Patch

Mrs Diane Simpson

Mrs Lee Tbaily

Dr Alicja A'Court

Dr James Bain

Ms Emma Barr

Dr Kate Blethyn

Dr Thomas Craig

Dr Hugh Cutler

Dr Jenny Davies

Dr Rebecca Fry

Mrs Elizabeth Hawes

Dr Nicholas Jenkins

Dr Martin Knight

Dr David Restall

Dr Matthew Stubbs

Dr Angus Sutherland

Dr Claire Swarbrick

Dr Robin Wilson

Dr Paul Winwright

Dr Thomas Dawes

Dr Ben Eden Green

Dr Julian Giles

Mrs Gail Pottinger

Ms Debbie Weller

Ms Lisa Burgess

Mrs Jean Denton

Ms Alaine Done

Mrs Jayne Edwards

Mrs Theresa Garrett

Mr Rajesh Gilla

Mrs Samantha Griffith-Norris

Dr John John

Ms Teresa Jones

Mrs Barbara Linklater-Jones

Ms Claire Nicholas

Miss Sara Owen

Miss Charlotte Perkins

Mrs Tessa Rowlands

Mrs Julie Steen

Miss Sarah Turner

Mrs Johanna Wales

Miss Carol-Ann Woolley

Dr Aruthy Arumugam

Ms Georgina Bird

Ms Nichola Bleasby

Dr Peter Chater-Lea

Ms Emma Craig

Dr George Davies

Dr Patrick Dill-Russell

Ms Julie Foxton

Dr Helen Gilfillan

Dr Keelan Jerram

Dr Søren Kudsk-Iversen

Dr Rebecca Lee

Dr John Livesy

Dr Amanda Mohabir

Dr Katherine Morris

Dr Mike Raffles

Dr Annika Smith

Dr Huiqi Wang

Miss Benita Adams

Mrs Sarah Bean

Dr Julian Berry

Miss Karen Burt

Miss Nicki Devooght-johnson

Miss Fiona Hammonds

Dr Tom Nicholls

Miss Michelle Rowe

Miss Jessica Summers

Dr Olivia Ward

Miss Belinda Wroath

Miss Gabbie Young

Dr Alex Dunn

Miss Sara Eddy

Ms Lynda Garcia

Dr James Garwood

Dr Adam Green

Ms Karin Gupwell

Ms Nicola Jones

Dr Cathryn Matthews

Dr Francesca Mazzola

Ms Evanna McEvoy

Dr Yang Ng

Dr Stephanie Pauling

Mrs Alison Potter

Ms Caroline Renton

Dr Adam Samways

Mrs Pauline Sibley

Ms Jenny Stead

Mr Kevin Thorpe

Dr Elizabeth Turner

Ms Michelle Walter

Mr Kevin Windsor

Dr Noor Elahi

Dr James Hilton

Dr Rajeev Jha

Dr Rupinder Kaur

Dr Victoria Male

Dr Manjeet Save

Dr Sophie Tang

Dr Thomas Urwin

Dr Ruoling Yan

Dr Nicolas Hooker

Dr Mayavan Abayalingam

Dr Richard Boulding

Dr Philip Coakley

Mr Filipe Helder

Dr Kunal Joshi

Dr Carlos Kidel

Dr Saqib Naji

Mr Manuel Pinto

Dr Megan Smith

Dr Emily Spence

Dr Marc Wittenberg

Dr Yohinee Rajendran

Dr Colin Williams

Dr Arjun Ardeshna

Dr Peter Bamford

Dr Sarah Hagyard

Dr Patrick Johnson

Dr Martina McMonagle

Dr Hayleigh Morris

Dr Thomas Murphy

Dr Stavros Papadopoulos

Dr Emma Simpson

Dr Jonathan Smith-Williams

Dr Vin Vyapury

Dr Paul Gunning

Dr Sophie Benoliel

Dr Matthew Dickinson

Mrs Nanci Doyle

Ms Cheryl Marriott

Ms Sarah Martin

Ms Laura Montague

Ms Kate Penhaligon

Dr Rita Saha

Dr Ramai Santhirapala

Dr Lushani Suntharanathan

Ms Nichola Wakeford

Dr Carly Webb

Dr Toby Winterbottom

Dr Matt Aldridge

Ms Clare Bolton-Hill

Prof Tim Cook

Dr Lesley Jordan

Dr Inthu Kangesan

Dr Laura Kettley

Dr Edward Mew

Dr James Penketh

Dr Eleonore Quinn

Dr Sam Scholes

Dr Mahindra Chincholkar

Dr Leanne Darwin

Ms Joy Dearden

Dr Adele Flowerdew

Ms Louise Harrison

Dr Robert Hartley

Ms Andrea Ingham

Dr Ben Jones

Ms Stephanie Lee

Dr Elizabeth Longdon

Ms Tracy Marsden

Ms Kirsty Meats

Ms Angiy Michael

Mr Drew Norwood-Green

Ms Victoria O'Loughlin

Dr Holly Owen

Dr Manju Patel

Ms Jane Perez

Dr Oliver Pratt

Ms Jessica Whiston

Ms Andrea Wood

Dr Chloe Billingham

Mrs Caroline Clarke

Dr Xantha Holmwood

Ms Melba Knighton

Dr Becky Sands

Dr Paul Stevens

Ms Sophia Strong-Sheldrake

Dr Matthew Taylor

Dr Simon Williams

Dr Elizabeth Brodier

Dr Ozerah Choudhry

Dr Bethany Fitzmaurice

Dr Santhana Kannan

Dr Miriam Namih

Dr Nalini Sethia

Dr Katy Smith

Dr Usman Choudhry

Dr Bharati Rajdev

Dr Mrutyunjaya Rao Rambhatla

Dr Swetha Rambhatla

Dr Stuart Reilley

Dr Rebecca Rice

Dr Rahul Wakhle

Dr Chris Walmsley

DR Kay Anne Mak

Dr Jonathan Hulme

Mr Michael Agyemang

Ms Sarah Bird

Dr Henry Elms

Dr Laura Hammon

Dr Christopher Harrison

Dr Claire Hirst

Ms Faith Kibutu

Ms Esme Marshall

Ms Leanne Milner

Ms Alexandra Mudd

Dr Matthew Needham

Dr James Nicholas

Dr Rob Penson

Dr Joel Perfitt

Dr Lorna Ryan

Ms Jennie Smith

Miss Samantha Strong

Dr John Whitaker

Dr Sarah Willcock

Miss Elizabeth Bell

Dr James Briscoe

Dr Andy Cruickshanks

Dr Jake Drinkwater

Mr Sam Eggleston

Ms Meredith Harris

Ms Kay Housley

Dr Krish Kapoor

Ms Beverly Kilner

Ms Emily King

Ms Emily Kirk

Mr Paolo Mazzone

Ms Janet Middle

Mr Adam Mitchell

Dr Becky Morris

Mr Paul Ogle

Mr Mark Pinkerton

Ms Mayeth Recto

Dr Maria Rehnstrom

Dr Catherine Riley

Dr Helen Thornley

Miss Bethany Tookey

Mr Daniel West

Mr Shay Willoughby

Dr Ian Wrench

Ms Riquella Abbott

Ms Lisa Bacon

Dr Paul Jones

Ms Angela Loughlin

Ms Helen Moore

Dr Samuel Passey

Dr Rebekah Rodgers

Dr Christopher Godden

Dr David Howe

Dr Jane Wright

Dr Paul Foley

SN Samantha Hagan

Dr Simon Marcus

Ms Kirsty Baillie

Ms Ella Buchanan

Mr Martyn Cain

Mrs Kerry Colling

Dr Raquel Duarte

Dr Chris Gibb

Dr Jeremy Henning

Dr Ryan Hynd

Dr Victoria Irvine

Dr Rob Lyons

Mrs Karoline Middleton

Dr Usman Razaque

Dr Faisal Sheikh

Dr Tariq Tabiner

Ms Carly Brown

Ms Gayle Clifford

Dr Christian Frey

Ms Madeleine McKee

Ms Beverley Stidolph

Sr Bridget Campbell

Ms Sophie Mason

Sr Penny Parsons

Dr Natasha Santana-Vaz

Ms Camilla Stagg

Dr Emert White

Ms Irene Gardner

Dr Christopher Goddard

Ms Zena Haslam

Dr David Morris

Ms Moira Morrison

Dr Chai Obeysekera

Dr Kim Porter

Ms Helen Terrett

Mrs Veronica Barnes

Dr Penny Bedoes

Dr Jonathan Clarke

Dr Robert Crichton

Dr Madhurima Das

Dr Paul Davies

Miss Helen Farrah

Dr Aoife Hegarty

Mrs Luisa Howlett

Dr Maren Kleine-Brueggeney

Dr Samson Ma

Dr Veronica Marsh

Dr Katherine McAndrew

Mr Johannes Mellinghoff

Dr Christopher Oscier

Mrs Christine Ryan

Dr Nirav Shah

Dr Elizabeth Smee

Dr Dushanthi Thurairasa

Dr Christopher Adeney

Dr Sanjay Behl

Ms Karen Chadwick

Dr Thomas Coleman

Dr Claire Davies

Mrs Sharon Dealing

Dr Jamie Elwood

Dr Emma Fadden

Dr Stella Gillies

Dr Vandana Goel

Dr Tom Kennedy

Dr Thomas Miller

Dr Simon Morton

Dr Rebecca Reilly

Dr Sue Smyth

Dr Thomas Syratt

Dr Emma Welfare

Ms Jennifer Awolesi

Dr Hannah Bennett

Dr Elizabeth Bradshaw

Dr David Crabtree

Ms Lauren Duraman

Ms Kerry Hughes

Ms Sarah Kirk

Ms Dee Leonard

Dr Rachel Stoeter

Dr Elizabeth Thomas

Dr Matyas Andorka

Dr Kingsin Ang

Mrs Linda Bailey

Dr Stephanie Brooks

Mrs Oon Chiu

Mrs Sally Collins

Dr James Jack

Mr Harry Knight

Miss Anastasia Lynn-Smith

Dr Matt Mackenzie

Dr Sarah Maher

Mrs Louise Nimako

Dr Simon Parrington

Miss Siva Sangaralingham

Dr Seliat Sanusi

Mrs Samantha Weller

Mrs Elizabeth Willard

Dr Peter Carroll

Mr Martyn Clark

Mrs Martina Coulding

Dr Elizabeth Denman

Dr Mohyman El Habishi

Dr Patrick Haywood

Dr Anand Kulkarni

Mrs Jane McConniffe

Ms Jacqueline McCormick

Dr Krishnakar Melachuri

Dr Evelyn Philip

Mrs Stephanie Ridgway

Mrs Heather Savill

Dr Kamal Sharif

Mrs Joanne Vere

Dr Alex Coombs

Ms Flora Darch

Mrs Patricia Doble

Mrs Jayne Foot

Dr Chris Gillett

Dr Stephen Harris

Dr Thomas Judd

Mrs Alison Moss

Miss Corinne Pawley

Mrs Moira Tait

Ms Alison Whitcher

Dr Natalie Wood

Dr Sam Clark

Dr Larysa Duniec

Dr Jaya Nariani

Dr Nipun Agarwal

Mrs Clare Allcock

Dr Irmeet Banga

Mrs Kirsty Baron

Mrs Andrea Cole

Dr Santinder Dalay

Mrs Ranjit Gidda

Dr Phillip Lo

Mrs Susan Merotra

Dr Kathryn Newton

Dr Richard Pierson

Dr Zoe Riddell

Dr Julian Sonksen

Mrs Sohan Bisonoothan

Dr Emma Casely

Mrs Carina Casey

Dr Elisa Kam

Mrs Geraldine Landers

Dr Constandinos Papageorgiou

Mrs Laura Pearse

Dr Meera Raja

Dr Dan Solomon

Dr Daniel Solomon

Dr Plamen Stoyanov

Dr Raheel Ahmed

Dr Rachael Bird

Dr David Buckley

Ms Verity Calder

Dr Caroline Clark

Ms Katherine Cullen

Mr John Davis

Dr Helen Doherty

Dr Bhaskar Dutta

Dr Matthew Faulds

Dr Jonathan Fortune

Ms Alexandra Gatehouse

Dr David Hay

Dr Michael James

Dr Karuna Kotur

Dr Peter Lawley

Dr Nimali Lochanie

Dr Sam Marcangelo

Ms Lesley McShane

Dr Amy Morgan

Dr Stephen Mowat

Dr Denis O'Leary

Dr Jennifer Partridge

Dr Janaki Pearson

Dr Sneha Prasad

Dr Girish Rangaswamy

Ms Carmen Scott

Dr Karen Smallshaw

Mr Graham Soulsby

Dr Anna Wahed

Ms Rebecca Wilson

Dr Claire-Marie Agius

Dr Sameer Ahmed

Dr Arjun Alva

Dr Emily Craven

Ms Sadie Diamond-Fox

Dr Jane Gibson

Dr Steve Hillier

Mr Paul Hindmarch

Mr Kiran Keshvara

Dr Lisa Macbeth

Dr Yamuna Madhu

Dr Joshua Nelson

Dr Tom Poulton

Dr Kay Protheroe

Dr Adnaan Qureshi

Ms Victoria Richardson

Dr Abdelrahman Soliman

Dr Iain Walker

Dr Sarah Welch

Dr Jessica Wilson

Dr Elaine Winkley

Dr Jonathan Womack

Mrs Anne Adams

Dr Orlanda Allen

Dr Marilyn Boampomaa

Mrs Anna-Marie Boniface

Dr Robert Coe

Mrs Amelia Daniel

Mrs Joanne Finn

Dr Issac Gill

Dr Huw Griffiths

Dr Joanna Hackney

Dr Kevin Hamilton

Dr Ruth Han

Dr Edward Hare

Dr Edward Rintoul

Mr Ervin Shpuza

Dr Ruth Smith

Mrs Nikki Staines

Dr Puvan Suppiah

Mrs Tracey White

Dr Ben Cracknell

Dr Nadine Farrell

Dr Jane Hermanowski

Ms Ruth Hodgson

Dr Toby Hoskins

Dr Rhiannon Jones

Dr Sam Keable

Dr Darcy Pearson

Mrs Melissa Rosbergen

Miss Dawn Collier

Miss Kathryn Dixon

Dr Michael Gardner

Professor Anil Hormis

Mrs Vicky Murray

Dr Harry Soar

Mrs Rachel Walker

Dr Shifa Yaruk

Dr Daniel Yarwood

Mrs Nina Barratt

Mr Andy Bates

Mr Norbert Bokor

Dr James Craig

Dr Julius Cranshaw

Mr Sergio Dominguez

Mrs Annette Fraine

Dr Richard Green

Mrs Katie Molloy

Miss Christina Penny

Mrs Sally Pitts

Miss Laura Purandare

Miss Taslima Rabbi

Miss Monica Serrano

Miss Tina Stoycheva

Mr Luke Vamplew

Mr Steve Williams

Ms Ethel Black

Dr Alex Eeles

Dr Justine Elliot

Dr Shaman Jhanji

Dr Rohit Juneja

Dr Reynard Knoetze

Dr Jamie McCanny

Dr Lauren Oswald

Dr Ellie Roderick

Dr Krishna Balachandar

Dr Chandra Bhimarasetty

Ms Laura Bird

Dr Conny Blunt

Dr Egidio Da Silva

Mr Mark Darbyshire

Ms Irene Echaveznaguicni

Dr Youssef Girgis

Dr Martin Goodman

Dr Dinkar Gowda

Dr Rajashekar Gowni

Ms Melony Hayes

Ms Natalie Jackson

Dr Anitha James

Dr Rekha Jayapal

Ms Claudette Jones

Ms Enid Leung

Dr Simran Minhas

Dr Mona Mubarak

Dr Sudeshkumar Muniyappa

Dr Zehrin Nassa

Dr Tom Neal

Dr Kudakwashe Nyangoni

Dr Suresh Panchakshariah

Ms Bethany Philpott

Mr Craig Pinner

Mr Daniel Pygall

Dr Manamohan Rangaiah

Dr Anil Rao

Dr William Rea

Dr Richard Shellard

Dr Guy Shinner

Dr Narendra Siddaiah

Dr Tony Sutherland

Dr Liza Tharakan

Ms Sue Thomas

Dr Betty Travasso

Dr Pyda Venkatesh

Ms Dionne Wortley

Dr Lara Allen

Sr Lyndsay Bibb

Sr Jill Brown

Miss Charlotte Busby

Sr Katherine Cheshire

Matron Beryl Davis

Mrs Rebecca Denyer

Sr Julie Edwards

Miss Laura Gardiner

Dr Shamim Haque

Miss Julia Icke

Miss Katherine Jones

Sr Donna McIntosh

Sr Mandy Oakley

Dr Linda Prasad

Sr Victoria Roberts

CN Andrew Smallwood

Sr Tammy Smith

CTA Lucy Stelfox

Sr Angela Stevens

Dr Sreekanth Uppugonduri

Sr Angela Willberry

Mrs Rashidat Adeniba

Dr Richard Armstrong

Ms Osi Egole

Dr Karen Fan

Ms Kayleigh Gilbert

Miss Laura Gould

Dr John Hickman

Ms Fei Long

Mr Sheik Pahary

Dr Michelle Reichman

Dr Jane Silk

Ms Kathryn Simpson

Ms Gabrielle De Selincourt

Ms Pauline Fitzell

Dr Katie Flower

Ms Kathleen Horan

Ms Bianca Hulance

Ms Pauline Mercer

Dr Jane Montgomery

Dr Sarah Shaw

Mr Robin Webber

Dr Seetal Aggarwal

Dr Amr Ali

Dr Boon Ang

Dr Lorna Filby

Dr Shannon Gawley

Ms Hindle Julia

Dr Manish Kakkar

Dr Robert Maher

Dr Samantha Moore

Dr Teodora Orasanu

Dr Louise Potter

Dr Niyesa Ranasinghe

Ms Thoy Ruth

Ms Butler Susie

Dr Tim Sutton

Dr Divya Veluvolu

Dr Charindri Wariyapola

Dr Lail Zaheer

Dr Vishnu Bhardwa

Mrs Wendy Deamer

Mrs Jo Fletcher

Dr Felix Fombon

Dr Chandrakant Gosavi

Dr Suganthi Joachim

Mrs Tara Lawrence

Mrs Kimberley Netherton

Dr Evangelia Poimenidi

Mr Lester Ribeiro

Ms Issy Thomas

Dr Thomas Walker

Dr Smita Bapat

Dr Gahan Bose

Dr Marta Campbell

Dr Preea Gill

Dr Astri Luoma

Dr Wael Zghaibe

Dr Catriona Ferguson

Dr Asya Mussad

Dr John Bugo

Dr Elizabeth Cervi

Dr Rachel Coathup

Dr Claire Frith

Dr Martin Gray

Dr Sachin Mehta

Dr Wint Mon

Dr Laura Parker

Dr Josh Patch

Dr Emily Robson

Dr Adrienne Stewart

Dr Filipe Vieira

Dr Josh Wall

Dr Jon Witby

Dr Sheldon Zhang

Dr Sarah Ciechanowicz

Dr Niveen El-Wahab

Dr Mayur Murali

Dr Paolo Perella

Dr Thunga Setty

Miss Elizabeth Bailey

Miss Roisin Baker

Ms Amy Bamford

Mr Colin Bergin

Miss Julia Blackburn

Dr Kinga Bodo

Mr Ronald Carrera

Miss Lauren Cooper

Miss Liesl Despy

Mrs Karen Ellis

Mr Morgan Foster

Mrs Sharon Garner

Mr Lyndon Harkett

Mrs Samantha Harkett

Mr Peter Ip

Dr Frances Lay

Mr HonSum Liu

Miss Laura Mee

Dr Samir Nazir

Mr Jacob Osbourne-Wylde

Miss Krupali Patel

Mr Martin Pope

Miss Emma Reeves

Miss Melanie Sahni

Dr Charlotte Small

Mr Brendon Spooner

Miss Elaine Spruce

Mrs Laura Tasker

Mr Arlo Whitehouse

Dr Sujata Anipindi

Dr Jennifer Aston

Mrs Joanne Bradley-Potts

Dr Ian Butler

Dr Jennifer Cunningham

Dr Alastair Duncan

Dr Hywel Garrard

Dr Daniel Haslam

Dr Danielle Kirk

Mr Richard McCormick

Dr Michael McEvoy

Dr Sara Mistry

Dr Danielle Ormandy

Dr Tracy Sharp

Dr Carolyn Smith

Dr Andrew Song

Dr Stephen Washington

Miss Georgina Williamson

Dr Francis Young

Dr Samar Al-Rawi

Dr Alice Aarvold

Dr Melissa Addy

Dr Georgina Ashfield

Dr Nigel Beauchamp

Dr Alex Belcher

Dr Tom Bennett

Dr Victoria Burgess

Dr Andy Burton

Dr Matthew Campbell

Dr Ross Cruikshank

Dr Andy Cumpstey

Dr Rhys Davies

Dr Chris Ford

Dr Renee Ford

Ms Lesley Hawkins

Dr Tim Hendra

Dr Thomas Huttley

Dr Erica Jolly

Ms Nevena Kalcheva

Dr Charlie Kennedy

Dr Jessica Lees

Dr Richard Lowe

Dr Tim Martindale

Dr Sarah McCormick

Dr Hannah McPhee

Dr Fran Millinchamp

Dr Rahul Muddanyake

Dr Eduardo Osorio

Dr Kiran Patel

Dr Samuel Pestell

Dr Steve Phillips

Dr Jamie Plumb

Dr Thomas Pratt

Dr Bert Quartermain

Dr Rebecca Reeves

Dr Sopihe Robin

Dr Thecla Scully

Dr Anamika Sehgal

Dr Said Seifalan

Dr Suzanne Shuttleworth

Dr Omar Siddique

Dr Esme Sleap

Dr Victoria Smith

Dr Richard Snooks

Dr Virginia Solanki

Dr Christina Timmons

Dr Alice Trimble

Dr Doug Tunney

Dr Mai Wakatsuki

Dr Kat Walker

Dr Naomi Wee

Dr James Wigley

Dr Huw Wilkins

Dr Hanna Wong

Dr Laura Wood

Dr Dinithi Yogya

Dr Ruth Young

Dr Rachel Alexander

Dr Amy Ashford

Dr Rebecca Aspinall

Dr Richard Bateman

Dr Matthew Bell

Ms Jennifer Bennett-Britton

Dr Jeremy Bewley

Dr Hannah Blanshard

Dr Christopher Bourdeaux

Dr Peter Brook

Dr Helen Cain

Dr Claire Cameron

Dr Naomi Cassells

Dr Nilesh Chauhan

Dr Adrian Clarke

Dr Melanie Cockroft

Dr Alan Cohen

Dr Joanna Collins

Dr Timothy Cominos

Dr Rachael Craven

Dr Ian Davies

Dr Sarah Dolling

Dr Claire Dowse

Kate Driver

Dr Adam Duffen

Dr Sarah-Jane Dunn

Dr Charlotte Earnshaw

Dr Frances Forrest

Dr Dan Freshwater-Turner

Dr Issie Gardner

Dr Matthew Gibbins

Dr Ben Gibbison

Dr Suzanne Gleeson

Dr Timothy Gould

Dr Matthew Govier

Dr Benjamin Gupta

Dr John Hadfield

Dr Nicola Harvey

Dr Sophia Henderson

Dr Lara Herbert

Dr James Hillier

Dr Elizabeth Hood

Dr Ben Howes

Dr Helen Howes

Dr Sian Hughes

Dr Kathryn Jackson

Dr Toby Jacobs

Dr Emma Jenkins

Dr Ian Jenkins

Dr Alistair Johnstone

Dr Lydia Jones

Dr Rebecca Jones

Dr Natasha Joshi

Dr Kajan Kamalanathan

Dr Neil Kellie

Dr Lawrence Kidd

Dr Mike (Stephen) Kinsella

Dr Thomas Knight

Dr Rebecca Leslie

Dr Stephen Linter

Dr Tim Lovell

Dr Matthew Martin

Dr Ursula McHugh

Dr Andrew McIndoe

Dr Rachel McKendry

Dr Alexander Middleditch

Dr Edward Miles

Dr Lesley Milne

Dr Mat Molyneux

Dr Neil Muchatuta

Dr Ruth Murphy

Dr Fran O’Higgins

Dr Kieran Oglesby

Dr Marcin Pachucki

Dr Claudia Paoloni

Dr Nishita Patel

Dr Catherine (Katie) Patton

Dr Skylar Paulich

Dr Annabel Pearson

Dr Anthony Pickering

Dr Martin Platt

Dr Charlie Pope

Dr Steve Pryn

Dr Amit Ranjan

Dr Neil Rasburn

Dr Andrew Ray

Dr Jonathan Rivers

Dr Kieron Rooney

Dr Ian Ryder

Dr Sarah Sanders

Dr Kavita Sasi-Kumar

Dr Liam Scott

Dr Mark Scrutton

Ms Chloe Searles

Dr James Self

Dr Sanjoy Shah

Dr Alison Shaw

Dr Mark Sheils

Dr Toby Shipway

Dr Heather Short

Dr Lauren Simmonds

Dr Daniela Smith

Dr Anjali Soodan

Dr Charlotte Steeds

Mrs Katie Sweet

Dr Phoebe Syme

Dr Matthew J Thomas

Dr Sally Tomkins

Dr Susan Underwood

Dr Adrian Wagstaff

Dr Paul Watson

Ms Denise Webster

Dr Katie Welham

Dr Anne Whaley

Dr Nicholas Wharton

Dr Helen Williams

Dr Hannah Wilson

Dr Anoushka Winton

Dr Thomas Woodward

Dr Jeanie Worthington

Dr Stuart Younie

Dr Mohammad Auldin

Dr Kate Bosworth

Dr Carol Bradbury

Dr Falguni Choksey

Dr Bernice Dudkowsky

Dr John Elton

Ms Gail Evans

Dr Charlotte Grove

Dr Clare Ingram

Dr Andrew Kelly

Dr Peter Lax

Dr Elena Lynes

Dr George Madden

Dr Vinesh Mistry

Dr Timothy Molitor

Ms Catherine Morgan

Ms Natalie Morris

Dr Emily Pallister

Ms Pooja Patel

Dr Vijay Ragothaman

Dr Katie Ramm

Dr Ben Robinson

Dr Meghna Sharma

Dr Daniel Shuttleworth

Dr Chandrashekhar Vaidyanath

Dr Jen Warren

Dr Rachel Wong

Ms Hannah Conway

Dr Gary Lau

Dr Adeel Majeed

Dr Jaina Parmar

Dr Meenal Rana

Dr Hakeem Yusuff

Dr Wael Abdelrhamen

Ms Kathryn Allison

Ms Karen Burns

Dr Nichola Cahill

Dr Jon Clark

Dr Hannah Donaldson

Dr Nick Heseltine

Ms Nicola Mackenzie

Ms Carol McArthur

Dr Neil Moreland

Dr Corinne Rimmer

Dr Sharmin Shohelly

Ms Hilary Thatcher

Dr Steven Tran

Dr Catriona Walker

Miss Nenette Abano

Miss Loretta Barnett

Mr Adrian Barry

Ms Pamela Birks

Ms Judith Brade

Mr Adrian Butler

Miss Racquel Carpio

Miss Viv Colclough

Ms Katrina Eaton

Ms Carol Edwards

Miss Lisa Emery

Ms Lisa Emery

Ms Kay Finney

Ms Susan Gallagher

Dr Satyajeet Ghatge

Miss Jeanette Grocott

Ms Amanda Hall

Ms Alison Hardwick

Miss Anne Harrison

Ms Susan Hendy

Miss Joanne Hiden

Dr Alex James

Dr Vijay Jeganath

Dr Rahul Kumar

Dr Stephen Lord

Ms Rachel Lovatt

Miss Susan Lyjko

Miss Holly Maguire

Ms Mia Marsden

Dr Venu Mehta

Dr Stephen Merron

Dr Rajeev Mishra

Dr Rohit Mittal

Mr Andrew Moores

Dr Pramod Nalwaya

Dr Matthew Omeara

Dr Sandor Orosz

Dr Nehal Patel

Ms Nicola Pattison

Miss Ida Ponce

Dr Ashok Puttapa

Dr Tamilselvan Rajamanickam

Dr Nagaraj Rao

Miss Alda Remegoso

Ms Rochelle Rhodes

Ms Angela Rooney

Ms Emma Sadler

Dr Permendra Singh

Dr Wai Soon

Dr Simon Tomlins

Dr Kanchan Umbarje

Dr Murali Vallabhaneni

Miss Resti Varquez

Ms Jill Wain

Dr Sorana White

Dr Elizabeth Willetts

Dr Benedict Williams

Dr Andrew Wood

Dr Farrukh Ameer

Dr Jonathan Finnity

Dr James Haddock

Dr Alex Hunt

Dr Aditya kuravi

Ms Fionnuala Lenehen

Dr Lindsay McOwat

Dr Asad Naqvi

Ms Lisa Richardson

Dr Sumant Shanbhag

Dr Victoria Siddons

Dr Deepa Jumani

Dr Daniel Zeinali

Dr Peter Bradley

Dr Laura Bubb

Mrs Rebekah Chan

Dr Seema Charters

Mrs Lynne Connell

Mrs Rachel Crone

Dr Hannah Davis

Mrs Pascal Defeyter

Dr Alison Evans

Dr Ramdas Howard

Mrs Helena Prady

Mrs Natalie Rogers

Mrs Lindsay Roughley

Mrs Helen Whittle

Mrs Jackie Evans

Dr Ann Lachana

Mrs Karen Markwell

Dr Ratna Makker

Dr Prasun Mukerjee

Dr Laura Osbourne

Dr Tomas Bakonyi

Dr Simon Ben-Nathan

Mrs Melanie Claridge

Dr Harriet Gardiner

Dr Ilya Kantsedikas

Mr Colin Merrill

Mrs Lynn O'Donohoe

Dr Fiona Oglesby

Dr Valerie Page

Dr Beena Parker

Miss Emelia Passaro

Dr Katherine Russell

Dr Sneh Shah

Mr Saul Sundayi

Mrs Elaine Walker

Ms Xiao Zhao

Dr Sajjad Ahmed

Dr Abdalla Ali

Dr Sarah Crawford

Ms Clare De'Ath

Dr Vijayakumar Gopal

Ms Sally Humphreys

Dr Carin Swanevelder

Dr Yeng Yap

Dr Vinanti Cherian McIvor

Dr Jonny Guy

Dr Emma O'Kane

Ms Sinead O'Kane

Dr Cathal Small

Dr Maqsood Bajwa

Dr Krzysztos Guz

Dr Nauman Iftikhar

Dr Beata Iwanicka

Dr Mujeeb Khan

Dr Muhammad Latif

Dr Anna-Marie Love

Dr Celia Montgomery

Dr Stephen Mulvany

Dr Mansoor Siddiqui

Dr Shaik Subhani

Miss Isobel Amey

Mrs Hilary Ashton

Mrs Yolanda Baird

Mrs Caroline Bushell

Dr Merle Cohen

Dr Emily Dana

Dr Emma Finlay

Ms Jeanette Gilbert

Dr Jenny Macallan

Dr Nic Martins

Dr Hannah Rose

Miss Indra Chadbourn

Dr David Helm

Dr Richard Kennedy

Mrs Carla Lewis

Mr Jordi Margalef

Ms Gabi Metiu

Mrs Sally Moore

Dr Tom Standley

Mrs Donna Cotterill

Dr Nicola Crowther

Mr Harvey Dymond

Dr Gurunath Hosdurga

Dr Thomas Ratcliffe-Law

Mr Glenn Saunders

Dr Thomas Saunders

Ms Fiona Brailsford

Dr Laura Bridge

Dr Lauren Collis

Ms Reni Jacob

Ms Cathy Jones

Ms Joanne Mullen

Dr Dabeeruddeen Ahmed

Dr Amanda Kirrage

Dr Jessica Lowe

Dr Andrew Prenter

Dr Sarbpreet Sarao

Dr David Sharpley

Dr Suresh Singaravelu

Dr Kavita Wankhade

Dr Caroline Davis

Dr Rachel Dolan

Dr Emma Pearson

Dr Cindy Persad

Dr Kate Blyth

Dr Andrew Burtenshaw

Dr Felicity Corcoran

Dr Nageena Hussain

Dr Anna Pierson

Dr Victoria Poyntz

Mrs Jessica Thrush

Dr Alex Wollaston

Mrs Julie Wollaston

Dr Paul Clements

Mrs Kirsty Duell

Mrs Linzi Heaton

Mrs Claire Hill

Mrs Ruth Killen

Mrs Nicola Pemberton

Dr Arindam Biswas

Dr Sarah Clayton

Dr Thomas Clayton

Dr Naomi Cochrane

Dr James Evans

Mr Mark Gaskell

Mrs Dianne Heaton

Mrs Sandra Latham

Dr Gemma McIntosh

Mr Chris Moore

Mrs Val Parkinson

Dr Matthew Robinson

Dr Alastair Sawyer

Mrs Isabelle Sykes

Mrs Tracey Taylor

Mrs Caroline Tierney

Mrs Katja van de Snepscheut-Jones

Miss Emily Wade

Dr Mohammed Wahid

Dr Henry Wang

Mrs Claire Williams

Dr Dominic Wu

Dr Manjula Yadagiri

Dr Dmitry Zabauski

Ms Eleanor Andrews

Dr Ranjit Bains

Dr Laura Beard

Ms Janine Birch

Dr Rachael Britton

Mr Mike Salmon

Dr Laura Troth

Ms Elizabeth Vassell

Mr Jamie Allen

Mrs Joanna Allison

Dr Andrew Baird

Dr Rob Gregory

Dr Agnieszka Kubisz-Pudelko

Dr Hannah Luckhurst

Dr Rebecca Mairs

Dr Ben Marshall

Mrs Lucy Pippard

Mrs Tressy Pitt-Kerby

Dr Andrew Savva

Dr Graham Walkden

Dr Celly Weegenaar

Mr Ben Chandler

Mrs Kerry Elliott

Ms Rosie Furness

Miss Laura Howe

Mrs Sarah Kent

Miss Alicia Rodgers

Mr Ben Straughan

Ms Emma Temlett

Dr Andy Chamberlain

Mr Simon Dyer

Mr Greg Forshaw

Mr Andy Gibson

Dr Muthuraj Kanakaraj

Mr John Whitwell

Miss Laura Farmer

Mr James Goodwin

Mr Jose Lourtie

Ms Sharon Drake

Dr James Bedford

Dr Oliver Boney

Dr Duncan Wagstaff

Dr David Gilhooly

Dr Maria Chazapis

**Australia**

Dr Ed O'Loughlin

Dr Andrew Emmanuel

Dr Cheryl Chooi

Dr Diyana Ishak

Dr Kellie Brick

Dr Liam Twycross

Dr Jannette Moss

Dr Essam Qazag

Dr Dave Gillespie

Dr Laurie Dwyer

Dr James Anderson

Dr Paige Bavich

Dr Ray Paramalingam

Dr Darren Sherwin

Dr Matthew Vendenburg

Dr Chelsea Hicks

Dr Stewart Anderson

Dr Armin Baghini

Dr Wilson Chee

Dr Anisha Kulkarni

Dr Rebecca Munk

Dr Lara Schemeczko

Dr Scott Popham

Dr Simone Fagan

Dr Anita Farmer

Dr Matthew Laraghy

Mr Angus Loraine

Dr Francia Van Der Merwe

Dr Winnie Yu

Dr Grigor Indjeian

Dr Helen Roberts

Dr Monica Diczbalis

Dr Gary Leung

Dr Matthew Spencer

Dr Aylin Spencer

Dr Dominik Teisseyre

Dr May Tsai

Dr Michelle Nguyen

Dr Heather Loane

Ms Leanne Pilkington

Dr Bronwyn Posselt

Dr Lillian Coventry

Dr Debra Leung

Dr David Shan

Dr David Highton

Dr Joshua Chong

Dr Ryan Droney

Dr Nathan Jeffrey

Mrs Allison Kearney

Dr Mary O'Shea

Dr Edward Piling

Dr Stephanie Pitt

Dr Justin Ti

Dr Maryann Turner

Dr Jonathan Wiggins

Dr Tony Miller-Greenman

Dr Raphael Weidenfeld

Dr Louis Guy

Dr Zenan Franks

Dr Elizabeth McLellan

Dr Angela Tognolini

Dr Gemma Pincus

Dr Sheridan Cohrs

Dr Courtney Thomas

Dr Madeleine Hanly

Dr Steven Klupfel

Dr Lucas Edwards

Dr Zoe Vella

Dr Thusira Karunaratne

Dr Andrew Lonergan

Dr Kathryn Meldon

Dr Tegan Burgess

Dr Vasheya Naidoo

Dr Georgina Prassas

Dr Erez Ben

Dr Joel Matthews

Dr Mitch Van Deurse

Dr Phoebe Lepper

Dr Annelise Kerr

Dr Libby Cawson

Miss Sophie Wallace

Professor Paul Myles

Dr Robert Smith

Dr Rachel Weir

Dr Nikjil Patel

Dr Edwin Kwan

Jennifer Preddy

**New Zealand**

Andrew Wilson

Lisa Barneto

Douglas Campbell

Helen Lindsay

Jonathan Panckhurst

Matthew Kirk-Jones

Karen Park

Everard Lee

Priya Shanmuganathan

Clare Ireland

Max Martensson

Dhir Bhattacharya

Han Truong

Jordan Tewhaiti-Smith

Kate Campbell

Vikramjit Singh

Sophie Gormack

Duncan Brown

Swarna Sharma

Alison Jackson

Tae Young Yoon

Qi Hao Ong

William White

Joe Follows

Sai Tim Yam

Prof Alexander (Sandy) Garden

Claire Smith

Tim Knowlman

Samuel Perrin

Jenny Henry

Nicole Vogts

Abhishek Charukonda
